# Differences in HIV cure clinical trial preferences of French people living with HIV and physicians in the ANRS‐APSEC study: a discrete choice experiment

**DOI:** 10.1002/jia2.25443

**Published:** 2020-02-20

**Authors:** Christel Protiere, Michael Arnold, Marion Fiorentino, Lisa Fressard, Jean D Lelièvre, Mohamed Mimi, François Raffi, Marion Mora, Laurence Meyer, Luis Sagaon‐Teyssier, David Zucman, Marie Préau, Olivier Lambotte, Bruno Spire, Marie Suzan‐Monti, the APSEC Study Group, J.F. Bergmann, J. Blacher, A.P. Blanc, P. Delobel, P.M. Girard, C. Goujard, C. Katlama, I. De Lacroix, A. Lafeuillade, J.D. Lelièvre, G. Lepeu, C. Michelet, J.M. Molina, P. Morlat, D. Peyramond, L. Piroth, I. Poizot‐Martin, F. Raffi, J.M. Ragnaud, E. Senneville, L. Weiss, Y. Yazdanpanh, D. Zucman

**Affiliations:** ^1^ INSERM, IRD, SESSTIM, Sciences Economiques & Sociales de la Santé & Traitement de l'Information Médicale Aix Marseille Univ Marseille France; ^2^ ORS PACA Observatoire régional de la santé Provence‐Alpes‐Côte d'Azur Marseille France; ^3^ Informing Change Berkeley CA USA; ^4^ INSERM Créteil France; ^5^ Faculté de médecine Université Paris Est Créteil France; ^6^ Vaccine Research Institute Créteil France; ^7^ Department of Infectious Diseases Hotel‐Dieu Hospital ‐ INSERM CIC 1413 Nantes University Hospital Nantes France; ^8^ Département d'épidémiologie, INSERM, U1018 Université Paris‐Sud 11 AP‐HP Hôpital de Bicêtre Le Kremlin‐Bicêtre France; ^9^ Hôpital Foch, service de médecine interne Suresnes France; ^10^ GRePS Lyon 2 Université Bron France; ^11^ Assistance Publique ‐ Hôpitaux de Paris Hôpital Bicêtre Service de Médecine Interne et Immunologie clinique Le Kremlin‐Bicêtre France; ^12^ Immunology of Viral Infections and Autoimmune Diseases INSERM, U1184 Le Kremlin‐Bicêtre France; ^13^ UMR 1184 Université Paris Sud Le Kremlin‐Bicêtre France; ^14^ CEA DSV/iMETI IDMIT Fontenay‐aux‐Roses France

**Keywords:** HIV eradication/remission, therapeutic HIV vaccine trial, social sciences, discrete choice experiment, preferences, ethics, clinical trial design recommendations, mixed logit model

## Abstract

**Introduction:**

Despite the advent of HIV cure‐related clinical trials (HCRCT) for people living with HIV (PLWH), the risks and uncertainty involved raise ethical issues. Although research has provided insights into the levers and barriers to PLWH and physicians' participation in these trials, no information exists about stakeholders' preferences for HCRCT attributes, about the different ways PLWH and physicians value future HCRCT, or about how personal characteristics affect these preferences. The results from the present study will inform researchers' decisions about the most suitable HCRCT strategies to implement, and help them ensure ethical recruitment and well‐designed informed consent.

**Methods:**

Between October 2016 and March 2017, a discrete choice experiment was conducted among 195 virally controlled PLWH and 160 physicians from 24 French HIV centres. Profiles within each group, based on individual characteristics, were obtained using hierarchical clustering. Trade‐offs between five HCRCT attributes (trial duration, consultation frequency, moderate (digestive disorders, flu‐type syndrome, fatigue) and severe (allergy, infections, risk of cancer) side effects (SE), outcomes) and utilities associated with four HCRCT candidates (latency reactivation, immunotherapy, gene therapy and a combination of latency reactivation and immunotherapy), were estimated using a mixed logit model.

**Results:**

Apart from severe SE – the most decisive attribute in both groups – PLWH and physicians made different trade‐offs between HCRCT attributes, the latter being more concerned about outcomes, the former about the burden of participation (consultation frequency and moderate SE). These different trades‐offs resulted in differences in preferences regarding the four candidate HCRCT. PLWH significantly preferred immunotherapy, whereas physicians preferred immunotherapy and combined therapy. Despite the heterogeneity of characteristics within the PLWH and physician profiles, results show some homogeneity in trade‐offs and utilities regarding HCRCT.

**Conclusions:**

Severe SE, not outcomes, was the most decisive attribute determining future HCRCT participation. Particular attention should be paid to providing clear information, in particular on severe SE, to potential participants. Immunotherapy would appear to be the best HCRCT candidate for both PLWH and physicians. However, if the risk of cancer could be avoided, gene therapy would become the preferred strategy for the latter and the second choice for the former.

## 1 Introduction

HIV cure research, a desired but risky and “uncomfortable” innovation 1, raises several ethical questions 2, 3, 4, 5, 6. More specifically, to test the effectiveness of new HIV cure‐related clinical trials (HCRCT), clinicians must recruit persons living with HIV (PLWH) who are successfully managing their health and the virus. Trial participation may have negative consequences for PLWH, including side effects (SE), increased transmission risk during antiretroviral treatment interruption (ATI) 7, disturbances to balanced lifestyles, and additional constraints and burdens related to the therapeutic strategies being tested. Currently, the expected benefit of participation is not remission or eradication, but an ATI 8, 9, 10 of only a few months for a low proportion of participants. This suggests a poor risk‐benefit ratio, which raises concerns about HCRCT participation coercion and consent 11.

The literature provides mixed findings regarding the willingness of PLWH to participate in HCRCT 12. In surveys, most respondents have expressed strong motivation and willingness 3, 13, 14. However, qualitative interviews indicate less interest 4, 15, 16, 17, 18. There may be several reasons for this, including differences in methodology and in questions posed, as well as discrepancies between declared intentions and actual behaviour 19.

HIV altruism is one important motivator influencing willingness to participate in HCRCT 3, 12, 15, 18, reflecting the historical advocacy and joint mobilization of PLWH in response to HIV epidemic 1, 2, 20, 21. Altruistic‐based benefits, such as participating in research for the benefit of future generations, have been shown to be part of the value associated with some health programmes 22, 23, 24. As a result, altruism should be considered a benefit induced by participation in HCRCT. Fear of SE and of the increased risk of transmission during ATI, the burden associated with appointments and clinical examinations, the poor expected personal clinical benefits, and uncertainty in terms of efficiency and SE, have all been shown to negatively influence willingness to participate 3, 12, 14, 15, 18. However, to the best of our knowledge, no study has investigated how stakeholders weigh different trial attributes when deciding about HCRCT participation.

Whenever possible, the type of HCRCT implemented should reflect PLWH trial preferences 13, 15, 25. Accordingly, a better understanding of how PLWH assess and make trade‐offs between different HCRCT attributes can provide useful insights about the best trial designs to implement, with a view to ensuring adequate numbers of participants to explore HCRCT effectiveness. Physicians 4, 12, 15, 18 and physician‐patient relationships 1, 26 can also influence PLWH motivations to participate. As physicians are not all equally motivated to propose HCRCT, it is important to also compare and contrast their preferences with those of PLWH 12, 14, 18.

An important role of social science is to inform real‐world decision making 27, 28. The cross‐sectional ANRS‐APSEC study, nested inside the IAS “Towards an HIV cure,” enabled us to explore, for the first time, the following three questions: (1) What trial design attributes most influence PLWH and physician trial acceptability? (2) To what extent do PLWH and physicians value candidate HCRCT differently? (3) How do differences within the PLWH and physician populations affect these preferences? Our results may help HCRCT researchers choose the most suitable HCRCT strategies to implement when designing trials, with a view to ensuring adequate participation numbers and effective HCRCT‐related communication strategies.

## 2 Methods

The survey received ethical approval from the CCTIRS (Advisory Committee on Information Processing of Research Information in the Field of Health) and the CNIL (National Commission for Computing and Liberties).

### 2.1 Survey population

Between October 2016 and March 2017, in 24 HIV centres across France, all HIV physicians and PLWH who had a follow‐up consultation during a dedicated week, and met eligibility criteria similar to those for inclusion in future HCRCT (≥18 years old, stable ART regimen ≥6 months, undetectable viral load ≥3 years, and a CD4 cell count >500 cells/mm^3^) were invited to participate in a discrete choice experiment (DCE).

All respondents received an information letter and provided written consent. Trained interviewers administered a face‐to‐face computer‐assisted questionnaire.

### 2.2 Development of HCRCT attributes to be evaluated and the DCE surveys

The underlying assumptions in a DCE are that healthcare interventions can be described by their attributes (or characteristics) and that a respondent's valuation of an intervention depends on the level of each of these attributes 29, 30, 31, 32. In DCE, respondents are presented with several pairs of hypothetical designs. Each time a pair is presented, respondents choose the design they would prefer, according to attribute levels. For example, in our study, a hypothetical HCRCT design may have more severe side effects (SE) but the best outcome (the outcome being defined as a given duration of antiretroviral treatment interruption (ATI) for a given percentage of participants) compared with another design (Table S1). By presenting an appropriate number of comparisons, we were able to infer how much a given attribute influenced respondents' decisions.

Based on previous results from the ANRS‐APSEC study, the literature 10, 12, 18 and consensus between the physicians on the study's scientific committee, five HCRCT attributes, each with either two or three levels, were retained for the DCE (Table 1).

**Table 1:** Description of attributes and their levels

Attribute	Level	Label	Description
1. Trial duration	1	Short	6‐9 months
	2	Long	15 to 18 months
2. Consultation frequency	1	Weekly	Weekly
	2	Monthly	Monthly
3. Moderate SE (1% to 10%, few days)	1	Low	Digestive disorders (nausea, vomiting)
	2	Intermediate	Flu‐type syndrome (fever, shivers, stiffness, joint pain)
	3	High	Digestive disorders, flu‐type syndrome, fatigue
4. Severe SE (<1/1000)	1	Low	Allergy
	2	Intermediate	Allergy, infections
	3	High	Allergy, infections, risk of cancer a few years later (missing data for estimating how frequent the risk is)
5. Outcomes (ATI duration, % of success)	1	3 to 6 months, 5%	ATI of three to six months, for 5% of patients
	2	6 to 12 months, 10%	ATI of six to twelve months, for 10% of patients

SE, side effects.

To reduce respondent burden while ensuring maximum information, to estimate relative preference coefficients for all attributes and levels, we used a partial factorial design supplied in R software package *support. CEs* 33. This resulted in a d‐efficient design consisting of 13 pair comparisons 34, 35, 36 with an 83% D‐efficiency score. Each of the 13 fixed pairs were presented to respondents in a randomized order to avoid anchor bias. The choice set was presented in an unlabelled form to avoid bias due to social representations and to ensure attention was given to the attributes 37.

Apart from the DCE, the face‐to‐face questionnaire collected data on sociodemographic characteristics, experience with HIV and ART, previous experience with HIV clinical trial participation, viewpoint regarding HCRCT, and sources used to gather HIV information. The PLWH and physician questionnaires were identical except that the former included the post‐traumatic growth (PTG) scale 38, 39. Post traumatic disorder being associated with voluntary treatment interruption 40.

In collaboration with the French NGO AIDES, a pilot survey was conducted with 10 PLWH, resulting in some questions being removed or reworded. The DCE exercise was well understood and mobilized PLWH interest.

### 2.3 Statistical and econometrical analyses

To explore the possibility of sample bias, we compared PLWH in our sample with the 638 PLWH from the French representative national VESPA2 cohort 41, 42 who met the same inclusion criteria as ours, regarding gender, age, professional status, perceived financial situation, housing occupational status and educational level.

#### 2.3.1 PLWH and physician profiles

To elicit distinct intragroup respondent profiles for PLWH and physicians, two hierarchical clustering procedures were performed on all variables (Table S2), except those related to DCE, using the SAS (v9.4), SAS Campus Drive, Cary, NC CAHQUAL macro‐command, developed by the French National Institute for Statistics and Economic Studies 43. We used the Ward method based on minimum inertia lost. The optimal number of clusters was determined with dendrograms, line plots of the cubic clustering criterion, and line plots of combined pseudo‐F and pseudo t^2^ statistics.

#### 2.3.2 Discrete choice experiment

We tested several models for the conjoint analysis of the DCE. First, a conditional logit model 44, which assumes common preferences for observed attributes; second, a mixed logit (MXL) model 45, which accounts for heterogeneity in the trade‐offs between the attribute levels but assumes that preferences between attributes are independent; third, a MXL model which accounts for heterogeneity and correlation in unobserved factors (captured by the error terms). This model was chosen based on Akaike's information criterion (AIC) 46 (results not shown). Analyses were performed using the *clogit* and *mixlogit* commands from Stata/SE 12.1 (Stata Corp LP, College Station, TX).

Two DCE models were estimated, one each for PLWH and physicians (intergroup comparison). To test for preference stability according to differences in respondent characteristics, two additional DCE models were estimated among PLWH and physician profiles (intragroup comparison). Measurement of the utility (value) associated with candidate HCRCT.

Although one would expect the preferred choice to be a hypothetical HCRCT design promising the fewest severe and moderate SE for the best outcome (six to twelve months ATI in 10% of patients), such a trial cannot exist in real life. Physicians from the scientific committee described four candidate HCRCT aimed at achieving a functional cure (long‐term control of HIV in the absence of ART), as the only current sterilizing cure (elimination of all HIV‐infected cells) option is allogeneic bone marrow transplant 2, 5, 47, 48, 49, 50, 51, 52, 53, 54, 55, 56. We described the four HCRCT (latent reactivation, immunotherapy, gene therapy and a combination of latency reactivation and immunotherapy) according to attributes and levels (Table S3).

To compare the utility of these four HCRCT, we applied significant estimated coefficients to each one, which were associated with corresponding attribute levels obtained from the PLWH and physician MXL models. A linear transformation was used to associate the lowest utility (U = 0) to the hypothetically worst design (attribute levels having the lowest coefficients) and the highest utility (U = 100) to the hypothetically best design (attribute levels having the highest coefficients). This linear transformation allowed us to compare the four HCRCT, according to their utility, on a 0 to 100 scale 30, 57, 58. 95% confidence intervals were estimated using the delta method 59, 60.

## 3 Results

### 3.1 Characteristics of the surveyed population

All the 195 PLWH (median inclusion = 9/centre) presented to the interviewers agreed to participate in the survey. Three (1.84%) of the 163 physicians (72.8%, median inclusion = 6/centre) who agreed to participate were excluded because they did not complete the DCE exercise.

Women comprised 23.6% of the PLWH sample, with a median (IQR) age of 53 (45 to 61) years, and median experience with HIV of 17 (11 to 25) years. Most (69.7%) considered themselves to be part of the PLWH community, and 41.5% part of the LGBT (lesbian, gay, bisexual, transgender) community. Eighty percent (80.5%) declared their willingness to participate in a HCRCT based on their preferred hypothetical design, as identified by their responses in the DCE.

In contrast to VESPA2 PLWH who met the same criteria, our sample comprised fewer women (33.4% vs. 23.6%, *p* < 0.05) and fewer individuals with less than a high‐school diploma (51.7% vs. 38.5%, *p* < 0.01). No other differences were observed.

Women comprised 51.3% of the physician sample, with a median (IQR) age of 50 (40 to 57) years, and a median (IQR) experience with HIV of 20 (10 to 26) years. The large majority considered themselves to be part of the heterosexual community (80.6%). Almost all (97.5%) declared they would propose the HCRCT based on their preferred hypothetical design.

#### 3.1.1 PLWH and physician profiles (results of hierarchical clustering)

Three distinct profiles were elicited for both the PLWH and physicians using hierarchical clustering (Table S2), each having three levels of perception of the burden associated with HIV and ART, plus a financial and psychological vulnerability effect for PLWH profiles, and a generational effect for physician profiles.

##### PLWH profiles

The first PLWH profile, labelled “comfortable & confident,” represented nearly half of the sample (48%). It included a large proportion of financially comfortable PLWH who declared no impact of HIV on their life and no need of moral support, who considered themselves well informed about HIV scientific advances, but who had no previous knowledge of HCRCT. They were confident about the long‐term efficiency of current ART and about the possible availability of an HIV cure during their lifetime. They considered research as a way to build on previous generations' work and were motivated to participate in cure research under certain conditions.

The second profile labelled “moderate” represented 19% of the sample. It included a large proportion of PLWH who considered themselves part of the LGBT community. They felt very concerned about the fact that living with HIV implies living with a secret, the use of condoms, the difficulty of having to take treatment every day, and associated SE.

The third profile, labelled “vulnerable & unconfident” represented 33% of the sample and included large proportions of women, of people co‐infected with HCV, of people expressing financial and health vulnerabilities, and people reporting that HIV and ART negatively impacted their lives. They had poorer self‐esteem and a greater need of HIV‐related moral support. They were less confident about the long‐term efficiency of ART and the availability of an HIV cure during their lifetime.

##### Physician profiles

The first physician profile, labelled “engaged & patient‐centered,” represented half of the sample (53%) and was characterized by a large proportion of physicians engaged in HIV clinical trials in general and HCRCT in particular. They self‐defined as heterosexuals and HIV activists and considered that HIV and ART had a strong negative impact on their patients' lives.

The second and third profiles represented the remaining half of the sample and included a large proportion of physicians who self‐reported being part of the LGBT community. They were split between the youngest and least experienced physicians (labelled “least experienced & moderate,” 29%) and the oldest and most experienced physicians (labelled “most experimented & reticent,” 19%). The latter profile more often expressed reticence toward clinical trials in general, and did not believe that ART or HIV induced great difficulties in their patients' lives. Those in the second profile adopted an intermediate position between the first and the third profiles.

### 3.2 Most decisive attributes in the decision to participate in/propose HCRCT (results from the DCE models)

#### 3.2.1 Intergroup comparison

**Figure 1:**
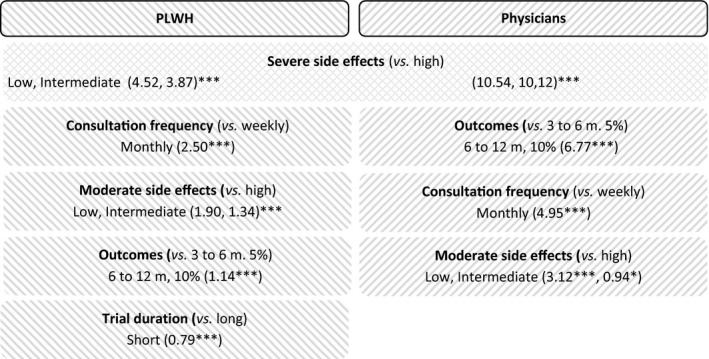
Results of the DCE for PLWH and physicians. ***p < 0.001, **p < 0.05, *p < 0.1.

Figure 1 shows that the most decisive attribute in the decision to participate in/propose HCRCT was the level of severe SE: the lower the level, the higher the willingness to participate/propose (Table S4). Furthermore, severe SE were more important for physicians (allergy: 10.54; allergy, infection: 10.12; *p* < 0.001) than for PLWH (allergy: 4.52; allergy, infections: 3.87; *p* < 0.001). The least decisive attribute for both was trial duration (a short duration being preferred), which was significant only for PLWH (0.79, *p* < 0.001).

With regard to the three other attributes, trade‐offs differed between the PLWH and physicians.

For PLWH, the second most decisive attribute was consultation frequency (monthly being preferred: 2.50, *p* < 0.001), followed by moderate SE (digestive disorders: 1.90, flu‐type syndrome: 1.34; *p* < 0.001) and then outcomes (ATI of six to twelve months, for 10% of patients: 1.14, *p* < 0.001).

For physicians, the second most decisive attribute was outcomes (6.77, *p* < 0.001) followed by consultation frequency (4.95, *p* < 0.001), and then moderate SE (3.12, *p* < 0.001).

#### 3.2.2 Intragroup comparisons

##### Between PLWH profiles

**Figure 2:**
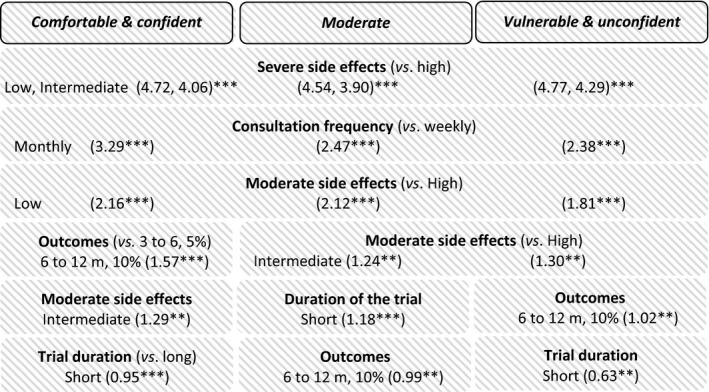
Results of the DCE for the three PLWH profiles. ***p < 0.001, **p < 0.05, *p < 0.1.

For all three PLWH profiles, the three most decisive attributes were severe SE (low and intermediate), followed by consultation frequency, and moderate SE (low) (Figure 2).

The main difference between profiles was the importance given to outcomes, which was a more decisive attribute for the *comfortable & confident* (1.57, *p* < 0.001) than for the *vulnerable & unconfident* (1.02, *p* = 0.007) and *moderate* (0.99, *p* = 0.013) PLWH. For the *comfortable & confident* and *vulnerable & unconfident* PLWH, trial duration was the least decisive attribute, whereas for *moderate* PLWH it was outcomes.

##### Between physician profiles

**Figure 3:**
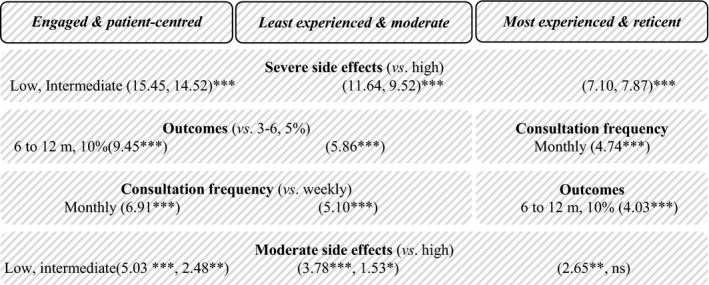
Results of the DCE for the three physician profiles. ***p < 0.001, **p < 0.05, *p < 0.1.

For all physician profiles, the most significant decisive attribute was severe SE while the least significant was moderate SE. The second most decisive attribute for the *most experienced & reticent* physicians was consultation frequency, whereas for the other two profiles it was outcomes (Figure 3).

### 3.3 Utilities associated with the four candidate HCRCT

#### 3.3.1 HCRCT utilities for PLWH and physicians

**Figure 4:**
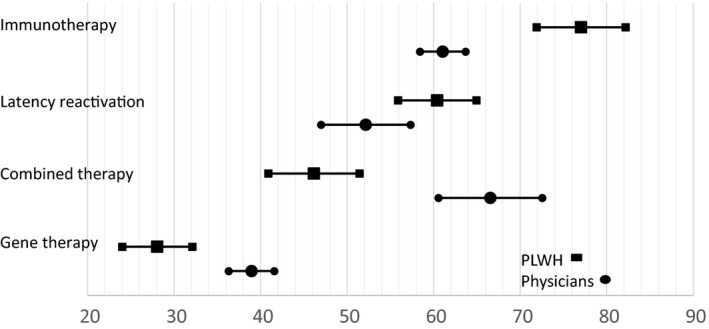
Utilities of four candidate HCRCT according to PLWH and physicians.

For PLWH, the order of preference was immunotherapy (77.03 (95% CI 71.89 to 82.17)), latency reactivation (60.41 (55.89 to 64.93)), combined therapy (46.16 (40.89 to 51.43)) and gene therapy (28.05 (24.00 to 32.10)), with significant differences between all four candidate HCRCT (Figure 4).

For physicians, combined therapy (66.55 (60.56 to 72.55)) and immunotherapy (61.05 (58.43 to 63.68)) were significantly preferred to latency reactivation (52.16 (46.99 to 57.33)), which in turn was significantly preferred to gene therapy (38.95 (28.65 to 40.60)).

Gene therapy implied the highest level of severe SE, including a risk of cancer within a few years for an unknown number of cases (Table S3). Consequently, it was significantly less preferred, irrespective of the population considered, and despite physicians valuing it significantly higher than PLWH. We then modelled gene therapy considering the intermediate level of severe SE (allergy, infections), instead of the highest level (allergy, infections, risk of cancer). In this case, gene therapy became the most preferred HCRCT for the physicians (78.8 (73.8 to 83.9)) and the second most preferred for the PLWH (63.7 (58.2 to 69.1)).

#### 3.3.2 HCRCT utilities for each of the PLWH and physician profiles

**Figure 5:**
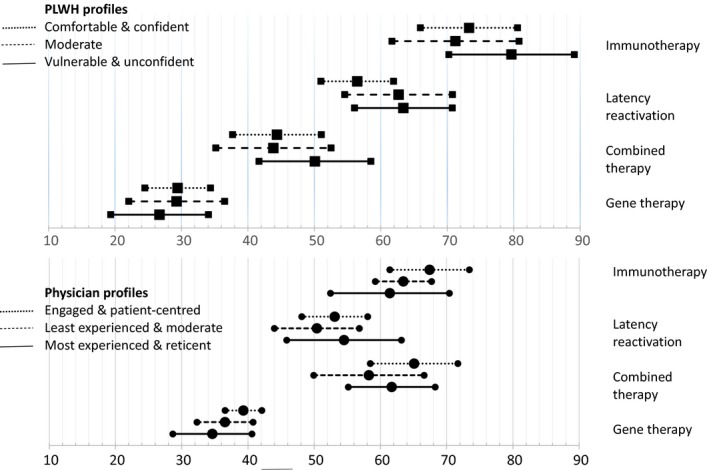
Utilities of four candidate HCRCT according to PLWH and physician profiles.

The preference order for the four candidate HCRCT was similar for the three intragroup profiles for both PLWH and physicians, but differed in terms of intensity (Figure 5). For example, immunotherapy (71.24 (61.67 to 80.80)) and latency reactivation (62.66 (54.55 to 70.78)) were significantly preferred to combined therapy (43.82 (35.15 to 52.49)) and gene therapy (29.27 (22.05 to 36.49)) for the *moderate* PLWH profile. For the *vulnerable & unconfident* PLWH profile, immunotherapy (79.67 (70.23 to 89.12)), latency reactivation (63.38 (56.02 to 70.74)) and combined therapy were (50.08 (41.65 to 58.50)) significantly preferred to gene therapy (26.69 (19.33 to 34.05)).

Among physicians, the *most experienced & reticent* profile stood out as there was no significant difference between immunotherapy (61.42 (52.45 to 70.40)), combined therapy (61.69 (55.14 to 68.25)) and latency reactivation (54.50 (45.84 to 63.17)).

## 4 Discussion

Despite major reductions in HIV‐related mortality and morbidity thanks to antiretroviral treatment (ART), as yet no cure exists. The lack of universal access to ART, new infections, toxicities related to ART, and the need for life‐long treatment all highlight the huge importance of finding a cure. HIV cure research requires active participation of person living with HIV (PLWH) and their physicians. In this context, the ANRS‐APSEC survey allowed us to elicit the preferences of these stakeholders regarding four candidate HIV Cure Related Clinical Trial (HCRCT): immunotherapy, latency reactivation, a combination of the two former, and gene therapy.

Our results showed that PLWH and physicians made different trade‐offs between five HCRCT attributes. Despite *outcomes* (a given duration of antiretroviral treatment interruption for a given percentage of participants) being more important for physicians than for PLWH, it was not the most decisive attribute to participate in/propose a HCRCT. The most decisive attribute for both groups was *severe Side Effects (SE)*, having greater importance for physicians, probably because PLWH trusted them 12, 18, 26. The biggest difference in trade‐offs was that physicians were more concerned about *outcomes* whereas PLWH were more concerned about their quality of life (*consultation frequency* and *moderate SE*). In line with previous research 3, 12, 15, 18, this result might indicate that PLWH motivations to participate were more altruistic than individual. It also underlines the need for physicians to consider moderate SE and not only severe SE. Despite the heterogeneity of characteristics within the PLWH and physician profiles, trade‐offs they made were relatively homogeneous. However, the *comfortable & confident* PLWH profile placed greater importance on *outcomes* (reflecting the physician preference hierarchy) than the other two PLWH profiles. This may be explained by their better life conditions and less worry regarding HCRTC‐related constraints. On the contrary, the *most experienced & reticent* physician profile placed greater importance on *consultation frequency* (reflecting the PLWH preference hierarchy) than the other two physician profiles.

The differences in PLWH and physicians' trades‐offs resulted in different preferences regarding HCRCT. PLWH significantly preferred immunotherapy whereas physicians preferred combined therapy and immunotherapy. Consequently, immunotherapy seemed to be the best HCRCT candidate. On the contrary, gene therapy was the least preferred HCRCT, because of the associated risk of cancer, and probably also because of the uncertainty related to this risk. Aversion to uncertainty was observed in previous analyses 12, 18. In terms of the profiles, the order of preference for the HCRCT was similar for both PLWH and physicians.

### 4.1 Strengths and limitations

Our sample comprised not only men who have sex with men – which is the population most studied in previous surveys – but also women and heterosexuals meeting the clinical criteria for inclusion in HCRCT. However, women and those with less than a high‐school diploma are underrepresented compared to PLWH from VESPA2 41, 42. Unfortunately, we could not evaluate whether this was due to chance related to consultation scheduling), or due to physicians suggesting participation in HCRCT less often to these subgroups 49. The physician sample comprised professionals familiar with HIV research, but not necessarily HIV cure research. This enabled heterogeneous viewpoints to be considered.

In addition to the four candidate HCRCT selected in this survey, the results of the DCE allowed us to estimate preferences for any HCRCT. Of course the five trial attributes are not enough to document the complexity of an HCRCT. However, they were selected as they were identified as the most decisive attributes in the previous steps of the ANRS‐APSEC survey 12, 18. Another attribute – exchange of information before and during the trial – was seen to be important 12, but adding this additional attribute would have led to an overly complex factorial design, thereby necessitating a very large sample. Moreover if future studies consider sterilizing cure, another important attribute to include will be the difference between remission and cure.

## 5 Conclusions

Despite the innovative context of HIV cure research, we found that severe side effects rather than outcomes were the most important attribute for both PLWH and physicians when deciding to participate in/propose an HIV Cure Related Clinical Trial (HCRCT). As a result, particular attention should be paid to provide clear information to potential participants, in particular regarding severe side effects, and to provide feedback about the results during the trial.

Our results suggest that immunotherapy is the best candidate HCRCT for PLWH and physicians. However, if the risk of cancer could be avoided, gene therapy would become the preferred HCRCT for physicians and the second choice for PLWH. Future surveys should be conducted in real‐world situations to compare and contrast declared intention with real behaviour.

## Competing interests

The authors have no competing interests to declare.

## Authors' contributions

CP, MaM, OL, MSM and BS implemented this work. LF and MoM led the analysis under the supervision of LST and CP. The manuscript was written collaboratively between CP, MA, LF and OL with input from DZ, FR, JDL, LM, MF, MP and BS. MaM provided ongoing support to design and perform data collection. All authors have read and approved the final manuscript.

## Abbreviations

ART, antiretroviral treatment; ATI, antiretroviral treatment interruption; DCE, discrete choice experiment; HCRCT, HIV Cure Related Clinical Trial; HCV, hepatitis C virus; LGBT, lesbian, gay, bisexual, transgender; PLWH, person living with HIV; SE, side effects.

## Supporting information


**Table S1.** Example of one of the 13 pairs of design comparisons

**Table S2.** Detailed results of the hierarchical clustering

**Table S3.** Description of four HCRCT candidates according to attribute levels

**Table S4.** Detailed results of the discrete choice experiment
