# Supplementary material for: Differences in HIV cure clinical trial preferences of French people living with HIV and physicians in the ANRS‐APSEC study: a discrete choice experiment
Source: J Int AIDS Soc. 2020 Feb 20;23(2):e25443. doi: 10.1002/jia2.25443 (PMC7048214; doi:10.1002/jia2.25443)
Supplement: Supplementary file 1 — Table S1. Example of one of the 13 pairs of design comparisons [file JIA2-23-e25443-s001.docx]

# Table S1. Example of one of the 13 hypothetical paired comparisons

|  | Design E | Design A |
| --- | --- | --- |
| **Trial duration** | 15-18 months | 15-18 months |
| **Consultation frequency** | Monthly | Weekly |
| **Moderate side effects**  (1-10%, few days) | Flu-type syndrome *(fever, shivers, stiffness, joint pain)*  Digestive disorders *(nauseas, vomiting)*  Fatigue | Flu-type syndrome *(fever, shivers, stiffness, joint pain)* |
| **Severe side effects**  (<1/10,000) | Allergy  Infections | Allergy |
| **HCRCT outcomes**  (duration of interruption, rate of success) | 6-12 months  10 in 100 persons | 3-6 months,  5 in 100 persons |
