# Supplementary material for: Differences in HIV cure clinical trial preferences of French people living with HIV and physicians in the ANRS‐APSEC study: a discrete choice experiment
Source: J Int AIDS Soc. 2020 Feb 20;23(2):e25443. doi: 10.1002/jia2.25443 (PMC7048214; doi:10.1002/jia2.25443)
Supplement: Supplementary file 2 — Table S2. Detailed results of the hierarchical clustering [file JIA2-23-e25443-s002.docx]

Table S2: Results of hierarchical clustering

# Hierarchical clustering among PLWH

1: ***Comfortable & confident*** profile

2: ***Moderate*** profile

3: ***Vulnerable & unconfident*** profile

|  | **N=184** | **PROFILE** | | | **All** |
| --- | --- | --- | --- | --- | --- |
|  |  | **1 (48%)** | **2 (19%)** | **3 (33%)** |  |
| **Identity and sociodemographic characteristics** | | | | | |
| **You are** | | | | | |
| A man | 141 | 79,8 | 88,6 | 65,0 | 76,6 |
| A woman | 43 | 20,2 | 11,4 | 35,0 | 23,4 |
| **p-value : 0.0201** |  |  |  |  |  |
| **You have children or someone in your care** | |  |  |  |  |
| Yes | 38 | 21,4 | 5,7 | 28,3 | 20,7 |
| No | 146 | 78,7 | 94,3 | 71,7 | 79,4 |
| **p-value : 0.0309** |  |  |  |  |  |
| **Perceived financial situation** | |  |  |  |  |
| Finding it difficult or impossible to get by without debt | 38 | 21,4 | 11,4 | 25,0 | 20,7 |
| Just making ends meet | 60 | 23,6 | 34,3 | 45,0 | 32,6 |
| Quite comfortable | 48 | 23,6 | 37,1 | 23,3 | 26,1 |
| Very comfortable | 38 | 31,5 | 17,1 | 6,7 | 20,7 |
| **p-value : 0.0027** |  |  |  |  |  |
| **You are** | | |  |  |  |
| Owner | 74 | 41,6 | 54,3 | 30,0 | 40,2 |
| Tenant | 100 | 56,2 | 42,9 | 58,3 | 54,4 |
| Other | 10 | 2,3 | 2,9 | 11,7 | 5,4 |
| **p-value : 0.0291** |  |  |  |  |  |
| **Feel part of the LGBT community** | | | |  |  |
| Yes | 76 | 40,5 | 60,0 | 31,7 | 41,3 |
| No | 108 | 59,6 | 40,0 | 68,3 | 58,7 |
| **p-value : 0.0251** |  |  |  |  |  |
| **Score 0 (ashamed) - 10 (proud) scale** | | | |  |  |
| <=25th | 102 | 41,6 | 54,3 | 76,7 | 55,4 |
| 25-75th | 17 | 6,7 | 22,9 | 5,0 | 9,2 |
| >=75th | 65 | 51,7 | 22,9 | 18,3 | 35,3 |
| **p-value : <.0001** |  |  |  |  |  |
| **Score 0 (unconfident) – 10 (confident) scale** | | | | |  |
| <=25th | 52 | 13,5 | 34,3 | 46,7 | 28,3 |
| 25-75th | 80 | 40,5 | 51,4 | 43,3 | 43,5 |
| >=75th | 52 | 46,1 | 14,3 | 10,0 | 28,3 |
| **p-value : <.0001** |  |  |  |  |  |
| **Score 0 (sick) – 10 (healthy) scale** | | | | |  |
| <=25th | 49 | 10,1 | 17,1 | 56,7 | 26,6 |
| 25-75th | 85 | 43,8 | 74,3 | 33,3 | 46,2 |
| >=75th | 50 | 46,1 | 8,6 | 10,0 | 27,2 |
| **p-value : <.0001** |  |  |  |  |  |
| **Score 0 (excluded) à 10 (integrated) scale** | | | |  |  |
| <=25th | 46 | 9,0 | 14,3 | 55,0 | 25,0 |
| 25-75th | 61 | 25,8 | 68,6 | 23,3 | 33,2 |
| >=75th | 77 | 65,2 | 17,1 | 21,7 | 41,9 |
| **p-value : <.0001** |  |  |  |  |  |
| **Score 0 (vulnerable) - 10 (strong)** | | | | |  |
| <=25th | 55 | 10,1 | 20,0 | 65,0 | 29,9 |
| 25-75th | 75 | 40,5 | 74,3 | 21,7 | 40,8 |
| >=75th | 54 | 49,4 | 5,7 | 13,3 | 29,4 |
| **p-value : <.0001** |  |  |  |  |  |
| **Self-consider an HIV activist** | | | | | |
| Totally | 57 | *38,2* | 14,3 | 30,0 | 31,0 |
| Somewhat | 46 | 20,2 | *40,0* | 23,3 | 25,0 |
| Not really | 56 | 27,0 | *40,0* | 30,0 | 30,4 |
| Not at all | 25 | 14,6 | 5,7 | *16,7* | 13,6 |
| **p-value : 0.0580** | |  |  |  |  |
| **Age (years)** | | | | | |
| 23-44 | 46 | 22,5 | *37,1* | 21,7 | 25,0 |
| 45-52 | 42 | 22,5 | 20,0 | *25,0* | 22,8 |
| 53-60 | 50 | 27,0 | 20,0 | *31,7* | 27,2 |
| 61-84 | 46 | *28,1* | 22,9 | 21,7 | 25,0 |
| **p-value : 0.5973** | |  |  |  |  |
| **Marital status** |  |  |  |  |  |
| You are in a relationship and you live with your partner | 79 | 44,9 | 48,6 | 36,7 | 42,9 |
| You are in a relationship and you do not live with your partner | 19 | 13,5 | 5,7 | 8,3 | 10,3 |
| You are not in a relationship but you have sexual encounters | 47 | 24,7 | 22,9 | 28,3 | 25,5 |
| You are not in a relationship and you do not have sexual encounters | 39 | 16,9 | 22,9 | 26,7 | 21,2 |
| **p-value : 0.5839** | |  |  |  |  |
| **Feel part of the heterosexual community** | | | | | |
| Yes | 46 | 24,7 | 17,1 | 30,0 | 25,0 |
| No | 138 | 75,3 | 82,9 | 70,0 | 75,0 |
| **p-value : 0.3760** | |  |  |  |  |
| **Feel part of the PLWH community** | | | | | |
| Yes | 127 | 65,2 | 71,4 | 73,3 | 69,0 |
| No | 57 | 34,8 | 28,6 | 26,7 | 31,0 |
| **p-value : 0.5394** | |  |  |  |  |
| **You are professionally active** | |  |  |  |  |
| Yes | 105 | *61,8* | *65,7* | 45,0 | 57,1 |
| No | 79 | 38,2 | 34,3 | *55,0* | 42,9 |
| **p-value : 0.0656** | |  |  |  |  |
| **Your highest level of study is** | |  |  |  |  |
| Less than high-school diploma | 70 | 40,5 | 28,6 | 40,0 | 38,0 |
| High-school diploma | 40 | 25,8 | 20,0 | 16,7 | 21,7 |
| 2 years’ third-level education | 24 | 9,0 | 17,1 | 16,7 | 13,0 |
| 3 and more years’ third-level education | 50 | 24,7 | 34,3 | 26,7 | 27,2 |
| **p-value : 0.4814** | |  |  |  |  |
| **Clinical data, perception and evolution of health** | | | |  |  |
| **Self-perceived health state** | | | |  |  |
| Excellent | 29 | 27,0 | 0,0 | 8,3 | 15,8 |
| Very good | 56 | 40,5 | 37,1 | 11,7 | 30,4 |
| Good | 86 | 31,5 | 62,9 | 60,0 | 46,7 |
| Poor | 13 | 1,1 | 0,0 | 20,0 | 7,1 |
| **p-value : <.0001** |  |  |  |  |  |
| **Since HIV diagnosis, you consider that your health state** | | | | |  |
| Has improved | 67 | 44,9 | 28,6 | 28,3 | 36,4 |
| Has not changed | 83 | 50,6 | 51,4 | 33,3 | 45,1 |
| Has deteriorated | 34 | 4,5 | 20,0 | 38,3 | 18,5 |
| **p-value : <.0001** |  |  |  |  |  |
| **In the last 5 years, you consider your moral:** | | | | |  |
| Has improved | 61 | 40,5 | 28,6 | 25,0 | 33,2 |
| Has not changed | 104 | 57,3 | 62,9 | 51,7 | 56,5 |
| Has deteriorated | 19 | 2,3 | 8,6 | 23,3 | 10,3 |
| **p-value : 0.0008** |  |  |  |  |  |
| **Comorbidities: VHC** | |  |  |  |  |
| Yes | 20 | 10,1 | 0,0 | 18,3 | 10,9 |
| No | 164 | 89,9 | 100,0 | 81,7 | 89,1 |
| **p-value : 0.0205** |  |  |  |  |  |
| **Have you been depressed?** | | |  |  |  |
| Yes, during the last 12 months | 26 | 9,0 | 14,3 | 21,7 | 14,1 |
| Yes, before that | 51 | 23,6 | 31,4 | 31,7 | 27,7 |
| No, never | 107 | 67,4 | 54,3 | 46,7 | 58,2 |
| **p-value : 0.0954** | |  |  |  |  |
| **Were you diagnosed in 1996 or after?** | | |  |  |  |
| Yes | 125 | 65,2 | 82,9 | 63,3 | 67,9 |
| No | 59 | 34,8 | 17,1 | 36,7 | 32,1 |
| **p-value : 0.1068** | |  |  |  |  |
| **First ART in 1996 or after?** | | |  |  |  |
| Yes | 155 | 80,9 | 91,4 | 85,0 | 84,2 |
| No | 29 | 19,1 | 8,6 | 15,0 | 15,8 |
| **p-value : 0.3436** | |  |  |  |  |
| **Current ART: protease inhibitor** | | |  |  |  |
| Yes | 47 | 19,1 | 37,1 | 28,3 | 25,5 |
| No | 137 | 80,9 | 62,9 | 71,7 | 74,5 |
| **p-value : 0.0971** | |  |  |  |  |
| **Current ART: integrase inhibitor** | | |  |  |  |
| Yes | 79 | 46,1 | 28,6 | 46,7 | 42,9 |
| No | 105 | 53,9 | 71,4 | 53,3 | 57,1 |
| **p-value : 0.1616** | |  |  |  |  |
| **Nadir>200** | | | |  |  |
| Yes | 122 | 66,3 | 68,6 | 65,0 | 66,3 |
| No | 62 | 33,7 | 31,4 | 35,0 | 33,7 |
| **p-value : 0.9388** | |  |  |  |  |
| **Number of comorbidities [0;4]** | |  |  |  |  |
| 0 | 98 | 49,4 | *74,*3 | 46,7 | 53,3 |
| 1 | 57 | *33,7* | 20,0 | *33,3* | 31,0 |
| 2+ | 29 | *16,9* | 5,7 | *20,0* | 15,8 |
| **p-value : 0.0807** | |  |  |  |  |
| **Comorbidities : VHB** | |  |  |  |  |
| Yes | 11 | 6,7 | 2,9 | 6,7 | 6,0 |
| No | 173 | 93,3 | 97,1 | 93,3 | 94,0 |
| **p-value : 0.6875** | |  |  |  |  |
| **Post-traumatic Growth Inventory Score** | | | |  |  |
| <=25th | 47 | 24,7 | 37,1 | 20,0 | 25,5 |
| 25-75th | 88 | 41,6 | 45,7 | 58,3 | 47,8 |
| >=75th | 49 | 33,7 | 17,1 | 21,7 | 26,6 |
| **p-value : 0.0864** | |  |  |  |  |
| **How much are you concerned about the following HIV-related difficulties?** | | | | | |
| **Shorter expected life** | | | | | |
| Very concerned | 61 | 22,5 | 20,0 | 56,7 | 33,2 |
| A little concerned | 57 | 22,5 | 60,0 | 26,7 | 31,0 |
| Not at all concerned | 66 | 55,1 | 20,0 | 16,7 | 35,9 |
| **p-value : <.0001** |  |  |  |  |  |
| **Severe fatigue** | | | | | |
| Very concerned | 43 | 3,4 | 14,3 | 58,3 | 23,4 |
| A little concerned | 61 | 25,8 | 60,0 | 28,3 | 33,2 |
| Not at all concerned | 80 | 70,8 | 25,7 | 13,3 | 43,5 |
| **p-value : <.0001** |  |  |  |  |  |
| **A negative impact on health** | | | | | |
| Very concerned | 42 | 5,6 | 5,7 | 58,3 | 22,8 |
| A little concerned | 58 | 21,4 | 62,9 | 28,3 | 31,5 |
| Not at all concerned | 84 | 73,0 | 31,4 | 13,3 | 45,7 |
| **p-value : <.0001** |  |  |  |  |  |
| **An uncertain future due to HIV** | | | | | |
| Very concerned | 68 | 18,0 | 20,0 | 75,0 | 37,0 |
| A little concerned | 58 | 28,1 | 57,1 | 21,7 | 31,5 |
| Not at all concerned | 58 | 53,9 | 22,9 | 3,3 | 31,5 |
| **p-value : <.0001** |  |  |  |  |  |
| **The risk of transmitting the disease** | | | | | |
| Very concerned | 89 | 34,8 | 45,7 | 70,0 | 48,4 |
| A little concerned | 40 | 21,4 | 40,0 | 11,7 | 21,7 |
| Not at all concerned | 55 | 43,8 | 14,3 | 18,3 | 29,9 |
| **p-value : <.0001** |  |  |  |  |  |
| **Discrimination due to HIV** | | | | | |
| Very concerned | 68 | 24,7 | 28,6 | 60,0 | 37,0 |
| A little concerned | 44 | 16,9 | 45,7 | 21,7 | 23,9 |
| Not at all concerned | 72 | 58,4 | 25,7 | 18,3 | 39,1 |
| **p-value : <.0001** |  |  |  |  |  |
| **Difficulties constructing a stable couple relationship** | | | | | |
| Very concerned | 54 | 21,4 | 22,9 | 45,0 | 29,4 |
| A little concerned | 43 | 14,6 | 42,9 | 25,0 | 23,4 |
| Not at all concerned | 87 | 64,0 | 34,3 | 30,0 | 47,3 |
| **p-value : <.0001** |  |  |  |  |  |
| **Having to live with a secret** | | | | | |
| Very concerned | 81 | 33,7 | 48,6 | 56,7 | 44,0 |
| A little concerned | 55 | 29,2 | 28,6 | 31,7 | 29,9 |
| Not at all concerned | 48 | 37,1 | 22,9 | 11,7 | 26,1 |
| **p-value : 0.0086** |  |  |  |  |  |
| **Feeling unable to lead a normal life** | | | | | |
| Very concerned | 43 | 14.6 | 14.3 | 41.7 | 23.4 |
| A little concerned | 57 | 20.2 | 42.9 | 40.0 | 31.0 |
| Not at all concerned | 84 | 65.2 | 42.9 | 18.3 | 45.7 |
| **p-value** |  |  |  |  |  |
| **A negative impact on sexuality** | | | | | |
| Very concerned | 72 | 29,2 | 37,1 | 55,0 | 39,1 |
| A little concerned | 49 | 22,5 | 25,7 | 33,3 | 26,6 |
| Not at all concerned | 63 | 48,3 | 37,1 | 11,7 | 34,2 |
| **p-value : 0.0002** |  |  |  |  |  |
| **Having to use condoms** | | | | | |
| Very concerned | 87 | 37,1 | 51,4 | 60,0 | 47,3 |
| A little concerned | 40 | 20,2 | 22,9 | 23,3 | 21,7 |
| Not at all concerned | 57 | 42,7 | 25,7 | 16,7 | 31,0 |
| **p-value : 0.0137** |  |  |  |  |  |
| **A negative impact on professional life due to HIV** | | | | | |
| Very concerned | 41 | 4,5 | 8,6 | 56,7 | 22,3 |
| A little concerned | 28 | 11,2 | 28,6 | 13,3 | 15,2 |
| Not at all concerned | 115 | 84,3 | 62,9 | 30,0 | 62,5 |
| **p-value : <.0001** |  |  |  |  |  |
| **The cost for the society** | | | | | |
| Very concerned | 91 | 44,9 | 40,0 | 61,7 | 49,5 |
| A little concerned | 43 | 19,1 | 45,7 | 16,7 | 23,4 |
| Not at all concerned | 50 | 36,0 | 14,3 | 21,7 | 27,2 |
| **p-value : 0.0016** |  |  |  |  |  |
| **Out-of pocket-expenses** | | | | | |
| Very concerned | 39 | 12,4 | 2,9 | 45,0 | 21,2 |
| A little concerned | 49 | 21,4 | 51,4 | 20,0 | 26,6 |
| Not at all concerned | 96 | 66,3 | 45,7 | 35,0 | 52,2 |
| **p-value : <.0001** |  |  |  |  |  |
| **Moral support availability** | | | | | |
| <=25th | 28 | 11,2 | 8,6 | 25,0 | 15,2 |
| 25-75th | 31 | 7,9 | 37,1 | 18,3 | 16,9 |
| >=75th | 43 | 22,5 | 28,6 | 21,7 | 23,4 |
| Not concerned | 82 | 58,4 | 25,7 | 35,0 | 44,6 |
| **p-value : 0.0001** |  |  |  |  |  |
| **Perception of life with ART** | | |  |  |  |
| **How do you feel about your current ART?** | | |  |  |  |
| Very comfortable | 123 | 82,0 | 51,4 | 53,3 | 66,9 |
| Quite comfortable | 58 | 18,0 | 48,6 | 41,7 | 31,5 |
| Quite uncomfortable | 3 | 0,0 | 0,0 | 5,0 | 1,6 |
| **p-value : 0.0002** |  |  |  |  |  |
| **How concerned are you about the need to take ART every day?** | | | | | |
| Very concerned | 85 | 29,2 | 57,1 | 65,0 | 46,2 |
| A little concerned | 45 | 23,6 | 37,1 | 18,3 | 24,5 |
| Not at all concerned | 54 | 47,2 | 5,7 | 16,7 | 29,4 |
| **p-value : <.0001** |  |  |  |  |  |
| **How concerned are you about the side effects associated with your current ART?** | | | | | |
| Very concerned | 51 | 13,5 | 14,3 | 56,7 | 27,7 |
| A little concerned | 56 | 16,9 | 62,9 | 31,7 | 30,4 |
| Not at all concerned | 77 | 69,7 | 22,9 | 11,7 | 41,9 |
| **p-value : <.0001** |  |  |  |  |  |
| **How uncomfortable are the side effects associated with your current ART?** | | | |  |  |
| Inexistent | 88 | 73,0 | 37,1 | 16,7 | 47,8 |
| Not uncomfortable at all | 27 | 12,4 | 28,6 | 10,0 | 14,7 |
| A little uncomfortable | 46 | 11,2 | 28,6 | 43,3 | 25,0 |
| Quite uncomfortable | 23 | 3,4 | 5,7 | 30,0 | 12,5 |
| **p-value : <.0001** |  |  |  |  |  |
| **Since you started ART, have you experienced side effects?** | | | | | |
| No | 53 | 44,9 | 17,1 | 11,7 | 28,8 |
| Yes, minor or moderate side effects | 72 | 33,7 | 48,6 | 41,7 | 39,1 |
| Yes, severe side effects | 46 | 18,0 | 34,3 | 30,0 | 25,0 |
| Yes, very severe side effects | 13 | 3,4 | 0,0 | 16,7 | 7,1 |
| **p-value : <.0001** |  |  |  |  |  |
| **Since you started ART, how frequently have you experienced side effects?** | | | | | |
| No side effects | 53 | 44,9 | 17,1 | 11,7 | 28,8 |
| Rare | 35 | 22,5 | 17,1 | 15,0 | 19,0 |
| Sometimes | 52 | 13,5 | 42,9 | 41,7 | 28,3 |
| Often | 44 | 19,1 | 22,9 | 31,7 | 23,9 |
| **p-value : <.0001** |  |  |  |  |  |
| **Experience and perception of clinical trials and research** | | | | | |
| **For you, participating in a clinical trial helps advance the work done by previous generations?** | | | | | |
| Totally agree | 138 | 87,6 | 71,4 | 58,3 | 75,0 |
| Mostly agree | 40 | 10,1 | 28,6 | 35,0 | 21,7 |
| Mostly disagree | 6 | 2,3 | 0,0 | 6,7 | 3,3 |
| **p-value : 0.0008** |  |  |  |  |  |
| **Globally, regarding your participation in a clinical trial,** | | |  |  |  |
| You are reticent | 15 | 5,6 | 8,6 | 11,7 | 8,2 |
| It depends on the trial’s characteristics | 83 | 44,9 | 45,7 | 45,0 | 45,1 |
| You follow the advice of your physician | 82 | 46,1 | 45,7 | 41,7 | 44,6 |
| You are favorable | 4 | 3,4 | 0,0 | 1,7 | 2,2 |
| **p-value : 0.7823** | |  |  |  |  |
| **Have you already participated in a clinical trial?** | | | |  |  |
| No | 102 | 57,3 | 62,9 | 48,3 | 55,4 |
| Yes, once | 41 | 21,4 | 14,3 | 28,3 | 22,3 |
| Yes, several times | 41 | 21,4 | 22,9 | 23,3 | 22,3 |
| **p-value : 0.5504** | |  |  |  |  |
| **Benefiting from closer medical follow up, was/should be a motivation to participate in a clinical trial?** | | | | | |
| Yes | 105 | 60,7 | 57,1 | 51,7 | 57,1 |
| No | 79 | 39,3 | 42,9 | 48,3 | 42,9 |
| **p-value : 0.5524** | |  |  |  |  |
| **Benefiting from better treatment than your current treatment, was/should be a motivation to participate in a clinical trial?** | | | | | |
| Yes | 114 | 64,0 | 62,9 | 58,3 | 62,0 |
| No | 70 | 36,0 | 37,1 | 41,7 | 38,0 |
| **p-value : 0.7746** | |  |  |  |  |
| **Not wanting to disappoint your doctor** | | |  |  |  |
| Yes | 34 | *21,4* | 11,4 | 18,3 | 18,5 |
| No | 150 | 78,7 | *88,6* | 81,7 | 81,5 |
| **p-value : 0.4399** | |  |  |  |  |
| **Knowing people who would participate in the same trial** | | | |  |  |
| Yes | 29 | 16,9 | 17,1 | 13,3 | 15,8 |
| No | 155 | 83,2 | 82,9 | 86,7 | 84,2 |
| **p-value : 0.8201** | |  |  |  |  |
| **Helping in medical advances, was/should be a motivation to participate in a clinical trial?** | | | |  |  |
| Yes | 174 | 93,3 | 100,0 | 93,3 | 94,6 |
| No | 10 | 6,7 | 0,0 | 6,7 | 5,4 |
| **p-value : 0.2888** | |  |  |  |  |
| **Information** | | | | | |
| **Do you feel well-informed by your HIV physician about HIV scientific advances?** | | | | | |
| Yes | 167 | 96,6 | 85,7 | 85,0 | 90,8 |
| No | 17 | 3,4 | 14,3 | 15,0 | 9,2 |
| **p-value : 0.0288** |  |  |  |  |  |
| **Sources of information about HIV cure research:**  **The specialized clinical team following you for your disease** | | | | | |
| Yes | 158 | 88,8 | 85,7 | 81,7 | 85,9 |
| No | 26 | 11,2 | 14,3 | 18,3 | 14,1 |
| **p-value : 0.4750** | |  |  |  |  |
| **Your attending physician** | | | | | |
| Yes | 82 | 51,7 | 42,9 | 35,0 | 44,6 |
| No | 102 | 48,3 | 57,1 | 65,0 | 55,4 |
| **p-value : 0.1294** | |  |  |  |  |
| **The media** | | | | | |
| Yes | 103 | 55,1 | 48,6 | 61,7 | 56,0 |
| No | 81 | 44,9 | 51,4 | 38,3 | 44,0 |
| **p-value : 0.4498** | |  |  |  |  |
| **Scientific articles** | | | | | |
| Yes | 74 | 36,0 | 42,9 | 45,0 | 40,2 |
| No | 110 | 64,0 | 57,1 | 55,0 | 59,8 |
| **p-value : 0.5105** | |  |  |  |  |
| **Patient associations** | | | | | |
| Yes | 22 | 11,2 | 2,9 | 18,3 | 12,0 |
| No | 162 | 88,8 | 97,1 | 81,7 | 88,0 |
| **p-value : 0.0775** | |  |  |  |  |
| **Your friends/family** | | | | | |
| Yes | 24 | 15,7 | 20,0 | 5,0 | 13,0 |
| No | 160 | 84,3 | 80,0 | 95,0 | 87,0 |
| **p-value : 0.0645** | |  |  |  |  |
| **Persons with the same illness as you** | | | | | |
| Yes | 36 | 24,7 | 8,6 | 18,3 | 19,6 |
| No | 148 | 75,3 | 91,4 | 81,7 | 80,4 |
| **p-value : 0.1195** | |  |  |  |  |
| **Knowledge and viewpoints regarding HIV Cure trials** | | | | | |
| **How important is it for you to be cured of HIV one day?** | |  |  |  |  |
| Very important | 158 | 89,9 | 68,6 | 90,0 | 85,9 |
| Important | 23 | 9,0 | 31,4 | 6,7 | 12,5 |
| Quite unimportant | 3 | 1,1 | 0,0 | 3,3 | 1,6 |
| **p-value : 0.0033** |  |  |  |  |  |
| **Do you think your current ART will continue to be efficient?** | | | | | |
| Do not know | 54 | 23,6 | 20,0 | 43,3 | 29,4 |
| Yes, short term | 6 | 1,1 | 0,0 | 8,3 | 3,3 |
| Yes, medium term | 19 | 6,7 | 14,3 | 13,3 | 10,3 |
| Yes, long term | 105 | 68,5 | 65,7 | 35,0 | 57,1 |
| **p-value : 0.0008** |  |  |  |  |  |
| **Have you previous knowledge of HIV cure trials?** | | | | |  |
| Yes | 74 | 29,2 | 48,6 | 51,7 | 40,2 |
| No | 110 | 70,8 | 51,4 | 48,3 | 59,8 |
| **p-value : 0.0125** |  |  |  |  |  |
| **Do you think a cure will become available in your lifetime** | | | | | |
| Yes | 114 | 73,0 | 48,6 | 53,3 | 62,0 |
| No | 23 | 7,9 | 22,9 | 13,3 | 12,5 |
| NSP | 47 | 19,1 | 28,6 | 33,3 | 25,5 |
| **p-value : 0.0259** |  |  |  |  |  |
| **A high number of participating care centers would be a motivation for you to participate in a cure trial?** | | | | | |
| Yes, totally | 38 | 21,4 | 5,7 | 28,3 | 20,7 |
| Yes, somewhat | 39 | 22,5 | 25,7 | 16,7 | 21,2 |
| Not really | 57 | 21,4 | 60,0 | 28,3 | 31,0 |
| Not at all | 50 | 34,8 | 8,6 | 26,7 | 27,2 |
| **p-value : 0.0003** |  |  |  |  |  |
| **A large number of participating patients would be a motivation for you to participate in a cure trial?** | | | | | |
| Yes, totally | 48 | 30,3 | 2,9 | 33,3 | 26,1 |
| Yes, somewhat | 54 | 27,0 | 42,9 | 25,0 | 29,4 |
| Not really | 41 | 12,4 | 51,4 | 20,0 | 22,3 |
| Not at all | 41 | 30,3 | 2,9 | 21,7 | 22,3 |
| **p-value : <.0001** |  |  |  |  |  |
| **A direct clinical benefit would be a motivation for you to participate in a cure trial** | | | | | |
| Yes, totally | 79 | 50,6 | 20,0 | 45,0 | 42,9 |
| Yes, somewhat | 62 | 25,8 | 60,0 | 30,0 | 33,7 |
| Not really | 28 | 15,7 | 20,0 | 11,7 | 15,2 |
| Not at all | 15 | 7,9 | 0,0 | 13,3 | 8,2 |
| **p-value : 0.0023** |  |  |  |  |  |
| **Treatments already tested for other diseases would be a motivation for you to participate in a cure trial** | | | | | |
| Yes, totally | 33 | 21,4 | 8,6 | 18,3 | 17,9 |
| Yes, somewhat | 61 | 24,7 | 60,0 | 30,0 | 33,2 |
| Not really | 51 | 23,6 | 25,7 | 35,0 | 27,7 |
| Not at all | 39 | 30,3 | 5,7 | 16,7 | 21,2 |
| **p-value : 0.0013** |  |  |  |  |  |
| **Would you discuss your intention to participate in cure trials with associations?** | | | | | |
| Yes | 26 | 11,2 | 2,9 | 25,0 | 14,1 |
| No | 158 | 88,8 | 97,1 | 75,0 | 85,9 |
| **p-value : 0.0063** |  |  |  |  |  |
| **Agreeing with the “conditional participation and access for all” viewpoint** | | | | |  |
| <=25th | 58 | 29,2 | 25,7 | 38,3 | 31,5 |
| 25-75th | 66 | 30,3 | 65,7 | 26,7 | 35,9 |
| >=75th | 60 | 40,5 | 8,6 | 35,0 | 32,6 |
| **p-value : 0.0004** |  |  |  |  |  |
| **Agreeing with the “moderately motivated” viewpoint** | |  |  |  |  |
| <=25th | 55 | 38,2 | 31,4 | 16,7 | 29,9 |
| 25-75th | 60 | 20,2 | 42,9 | 45,0 | 32,6 |
| >=75th | 69 | 41,6 | 25,7 | 38,3 | 37,5 |
| **p-value : 0.0039** |  |  |  |  |  |
| **Would you discuss your intention to participate in cure trials with your principal partner/spouse?** | | | | | |
| Yes | 87 | 46,1 | 62,9 | 40,0 | 47,3 |
| No | 97 | 53,9 | 37,1 | 60,0 | 52,7 |
| **p-value : 0.0937** | |  |  |  |  |
| **Would you discuss your intention to participate in cure trials with your parents?** | | | | | |
| Yes | 28 | 10,1 | 25,7 | 16,7 | 15,2 |
| No | 156 | 89,9 | 74,3 | 83,3 | 84,8 |
| **p-value : 0.0870** | |  |  |  |  |
| **Would you discuss your intention to participate in cure trials with other members of your family?** | | | | | |
| Yes | 58 | 31,5 | 28,6 | 33,3 | 31,5 |
| No | 126 | 68,5 | 71,4 | 66,7 | 68,5 |
| **p-value : 0.8902** | |  |  |  |  |
| **Would you discuss your intention to participate in cure trials with friends?** | | | | | |
| Yes | 77 | 37,1 | 57,1 | 40,0 | 41,9 |
| No | 107 | 62,9 | 42,9 | 60,0 | 58,2 |
| **p-value : 0.1176** | |  |  |  |  |
| **Would you discuss your intention to participate in cure trials with work colleagues?** | | | | | |
| Yes | 16 | 9,0 | 14,3 | 5,0 | 8,7 |
| No | 168 | 91,0 | 85,7 | 95,0 | 91,3 |
| **p-value : 0.2983** | |  |  |  |  |
| **Would you discuss your intention to participate in cure trials with other people on the web?** | | | | | |
| Yes | 26 | 14,6 | 8,6 | 16,7 | 14,1 |
| No | 158 | 85,4 | 91,4 | 83,3 | 85,9 |
| **p-value : 0.5417** | |  |  |  |  |
| **Would you discuss your intention to participate in cure trials with healthcare professionals?** | | | | |  |
| Yes | 22 | 12,4 | 5,7 | 15,0 | 12,0 |
| No | 162 | 87,6 | 94,3 | 85,0 | 88,0 |
| **p-value : 0.3991** | |  |  |  |  |
| **Very close follow-up would be a motivation for you to participate in a cure trial** | | | | | |
| Yes, totally | 87 | 49,4 | 45,7 | 45,0 | 47,3 |
| Yes, somewhat | 57 | 28,1 | 42,9 | 28,3 | 31,0 |
| Not really | 27 | 13,5 | 11,4 | 18,3 | 14,7 |
| Not at all | 13 | 9,0 | 0,0 | 8,3 | 7,1 |
| **p-value : 0.4119** | |  |  |  |  |
| **Agreeing with the “most motivated” viewpoint** | |  |  |  |  |
| <=25th | 52 | 28,1 | 31,4 | 26,7 | 28,3 |
| 25-75th | 56 | 25,8 | 40,0 | 31,7 | 30,4 |
| >=75th | 76 | 46,1 | 28,6 | 41,7 | 41,3 |
| **p-value : 0.4420** | |  |  |  |  |
| **Agreeing with the “reticence and way of life” viewpoint** | |  |  |  |  |
| <=25th | 64 | 38,2 | 34,3 | 30,0 | 34,8 |
| 25-75th | 46 | 23,6 | 40,0 | 18,3 | 25,0 |
| >=75th | 74 | 38,2 | 25,7 | 51,7 | 40,2 |
| **p-value : 0.0647** | |  |  |  |  |

# Hierarchical clustering among physicians

P1: ***engaged & patient-centered*** profile

P2: ***least experienced & moderate*** profile

P3: ***most experienced & reticent*** profile

|  | **N=154** | **PROFILE** | | | **All** |
| --- | --- | --- | --- | --- | --- |
|  |  | **P1 (53%)** | **P2 (29%)** | **P3 (19%)** |  |
| **Identity and sociodemographic characteristics** | | |  |  |  |
| **You are** |  |  |  |  |  |
| A man | 75 | 43,2 | 52,3 | 58,6 | 48,7 |
| A woman | 79 | 56,8 | 47,7 | 41,4 | 51,3 |
| **p-value : 0.3097** | |  |  |  |  |
| **Age** |  |  |  |  |  |
| 29-40 | 38 | 6,2 | 75,0 | 0,0 | 24,7 |
| 41-49 | 35 | 33,3 | 11,4 | 10,3 | 22,7 |
| 50-56 | 41 | 39,5 | 2,3 | 27,6 | 26,6 |
| 57-74 | 40 | 21,0 | 11,4 | 62,1 | 26,0 |
| **p-value : <.0001** |  |  |  |  |  |
| **Marital status** |  |  |  |  |  |
| You are in a relationship and you live with your partner | 109 | 76,5 | 70,5 | 55,2 | 70,8 |
| You are in a relationship and you do not live with your partner | 15 | 3,7 | 18,2 | 13,8 | 9,7 |
| You are not in a relationship but you have sexual encounters | 14 | 9,9 | 4,6 | 13,8 | 9,1 |
| You are not in a relationship and you do not have sexual encounters | 7 | 7,4 | 0,0 | 3,5 | 4,6 |
| Refusal to answer | 9 | 2,5 | 6,8 | 13,8 | 5,8 |
| **p-value : 0.0198** |  |  |  |  |  |
| **Feel part of the LGBT community** | | | |  |  |
| Yes | 14 | 3,7 | 13,6 | 17,2 | 9,1 |
| No | 140 | 96,3 | 86,4 | 82,8 | 90,9 |
| **p-value : 0.0434** |  |  |  |  |  |
| **Feel part of the heterosexual community** | | | | | |
| Yes | 125 | 92,6 | 75,0 | 58,6 | 81,2 |
| No | 29 | 7,4 | 25,0 | 41,4 | 18,8 |
| **p-value : 0.0001** |  |  |  |  |  |
| **Self-consider an HIV activist** | | | | | |
| Totally | 73 | 55,6 | 40,9 | 34,5 | 47,4 |
| Somewhat | 65 | 38,3 | 52,3 | 37,9 | 42,2 |
| Not really | 16 | 6,2 | 6,8 | 27,6 | 10,4 |
| **p-value : 0.0067** |  |  |  |  |  |
| **You are a homeowner** | | |  |  |  |
| Yes | 109 | 84,0 | 45,5 | 72,4 | 70,8 |
| No | 45 | 16,1 | 54,6 | 27,6 | 29,2 |
| **p-value : <.0001** |  |  |  |  |  |
| **Professional characteristics** | | |  |  |  |
| **You obtained your diploma how many years ago? [years]** | | |  |  |  |
| 01-09 | 36 | 7,4 | 68,2 | 0,0 | 23,4 |
| 10-20 | 38 | 33,3 | 15,9 | 13,8 | 24,7 |
| 21-27 | 39 | 34,6 | 6,8 | 27,6 | 25,3 |
| 28-45 | 41 | 24,7 | 9,1 | 58,6 | 26,6 |
| **p-value : <.0001** |  |  |  |  |  |
| **You have worked in your current service for how many years? [1;41 years]** | | |  |  |  |
| <=25th | 40 | 13,6 | 63,6 | 3,5 | 26,0 |
| 25-75th | 73 | 54,3 | 29,6 | 55,2 | 47,4 |
| >=75th | 41 | 32,1 | 6,8 | 41,4 | 26,6 |
| **p-value : <.0001** |  |  |  |  |  |
| **Length of experience with HIV care** | | | |  |  |
| 01-09 | 38 | 4,9 | 77,3 | 0,0 | 24,7 |
| 10-20 | 32 | 32,1 | 6,8 | 10,3 | 20,8 |
| 20-26 | 46 | 40,7 | 6,8 | 34,5 | 29,9 |
| 27-36 | 38 | 22,2 | 9,1 | 55,2 | 24,7 |
| **p-value : <.0001** |  |  |  |  |  |
| **Average number of patients seen in your service per week** | | | | | |
| Between 1 and 20 | 87 | 44,4 | 72,7 | 65,5 | 56,5 |
| Between 21 and 50 | 53 | 44,4 | 25,0 | 20,7 | 34,4 |
| Over 50 | 14 | 11,1 | 2,3 | 13,8 | 9,1 |
| **p-value : 0.0115** |  |  |  |  |  |
| **Academic or involved in research** | | | |  |  |
| Yes | 59 | 35,8 | 52,3 | 24,1 | 38,3 |
| No | 95 | 64,2 | 47,7 | 75,9 | 61,7 |
| **p-value : 0.0426** |  |  |  |  |  |
| **Ideally, your professional activity would be:** | | | | |  |
| More care-orientated | 22 | 4,9 | 27,3 | 20,7 | 14,3 |
| More research-orientated | 17 | 8,6 | 18,2 | 6,9 | 11,0 |
| The same | 115 | 86,4 | 54,6 | 72,4 | 74,7 |
| **p-value : 0.0014** |  |  |  |  |  |
| **Participating in a local HIV network** | | | | | |
| Yes | 105 | 75,3 | 61,4 | 58,6 | 68,2 |
| No | 49 | 24,7 | 38,6 | 41,4 | 31,8 |
| **p-value : 0.1313** | |  |  |  |  |
| **Have you participated in prevention or information actions organized by HIV associations** | | | | | |
| Yes | 65 | 45,7 | 27,3 | 55,2 | 42,2 |
| No | 89 | 54,3 | 72,7 | 44,8 | 57,8 |
| **p-value : 0.0403** |  |  |  |  |  |
| **Experience with clinical trials** | | |  |  |  |
| **Number of clinical trials you have participated in** | | |  |  |  |
| 01-09 | 27 | 7,4 | 38,6 | 13,8 | 17,5 |
| 10-20 | 33 | 12,4 | 31,8 | 31,0 | 21,4 |
| 20-39 | 47 | 34,6 | 27,3 | 24,1 | 30,5 |
| 40-99 | 47 | 45,7 | 2,3 | 31,0 | 30,5 |
| **p-value : <.0001** |  |  |  |  |  |
| **Globally, you are reticent about participating in a clinical trial** | | | | | |
| No | 152 | 100,0 | 100,0 | 93,1 | 98,7 |
| Yes | 2 | 0,0 | 0,0 | 6,9 | 1,3 |
| **p-value : 0.0127** |  |  |  |  |  |
| **Globally, your participation depends of the trial’s characteristics** |  |  |  |  |  |
| No | 54 | 34,6 | 43,2 | 24,1 | 35,1 |
| Yes | 100 | 65,4 | 56,8 | 75,9 | 64,9 |
| **p-value : 0.2463** |  |  |  |  |  |
| **Globally, you follow the chef de service’s advice** |  |  |  |  |  |
| No | 125 | 79,0 | 72,7 | 100,0 | 81,2 |
| Yes | 29 | 21,0 | 27,3 | 0,0 | 18,8 |
| **p-value : 0.0110** |  |  |  |  |  |
| **Globally, you are favorable to participating in a clinical trial** |  |  |  |  |  |
| No | 131 | 86,4 | 84,1 | 82,8 | 85,1 |
| Yes | 23 | 13,6 | 15,9 | 17,2 | 14,9 |
| **p-value : 0.8731** | 152 | 100,0 | 100,0 | 93,1 | 98,7 |
| **Participating in a clinical research protocol helps advance the work of previous generations** | | | | | |
| Agree totally | 98 | 72,8 | 59,1 | 44,8 | 63,6 |
| Somewhat agree | 45 | 24,7 | 34,1 | 34,5 | 29,2 |
| Somewhat disagree | 11 | 2,5 | 6,8 | 20,7 | 7,1 |
| **p-value : 0.0074** |  |  |  |  |  |
| **You participated in a previous trial to advance research** | | | |  |  |
| Yes | 126 | 88,9 | 77,3 | 69,0 | 81,8 |
| No | 28 | 11,1 | 22,7 | 31,0 | 18,2 |
| **p-value : 0.0377** |  |  |  |  |  |
| **You were** |  |  |  |  |  |
| Other | 7 | 1,2 | 13,6 | 0,0 | 4,6 |
| Principal investigator | 24 | 22,2 | 6,8 | 10,3 | 15,6 |
| Investigator | 121 | 76,5 | 79,6 | 82,8 | 78,6 |
| Member of the scientific committee | 2 | 0,0 | 0,0 | 6,9 | 1,3 |
| **p-value : 0.0003** |  |  |  |  |  |
| **How was your general experience of that trial?** | | |  |  |  |
| Very positive | 54 | 43,2 | 27,3 | 24,1 | 35,1 |
| Quite positive | 97 | 56,8 | 72,7 | 65,5 | 63,0 |
| Quite negative | 3 | 0,0 | 0,0 | 10,3 | 2,0 |
| **p-value : 0.0016** |  |  |  |  |  |
| **Results of the trial were** | | |  |  |  |
| Very satisfactory | 46 | 33,3 | 20,5 | 34,5 | 29,9 |
| Satisfactory | 96 | 64,2 | 72,7 | 41,4 | 62,3 |
| Unsatisfactory | 12 | 2,5 | 6,8 | 24,1 | 7,8 |
| **p-value : 0.0015** |  |  |  |  |  |
| **Do you consider you were adequately informed of the results of the trial and any advances made?** | | | | | |
| Totally | 57 | 38,3 | 25,0 | 51,7 | 37,0 |
| Somewhat | 61 | 43,2 | 38,6 | 31,0 | 39,6 |
| Not enough | 36 | 18,5 | 36,4 | 17,2 | 23,4 |
| **p-value : 0.0685** | |  |  |  |  |
| **Do you consider you adequately informed your patients of the potential benefits of the trial?** | | | | | |
| Totally | 71 | 54,3 | 40,9 | 31,0 | 46,1 |
| Somewhat | 79 | 42,0 | 59,1 | 65,5 | 51,3 |
| Not enough | 4 | 3,7 | 0,0 | 3,5 | 2,6 |
| **p-value : 0.1111** | |  |  |  |  |
| **Do you consider you adequately informed your patients of the potential risks of the trial?** | | | | | |
| Totally | 67 | 51,9 | 34,1 | 34,5 | 43,5 |
| Somewhat | 78 | 42,0 | 63,6 | 55,2 | 50,7 |
| Not enough | 9 | 6,2 | 2,3 | 10,3 | 5,8 |
| **p-value : 0.1116** | |  |  |  |  |
| **At the end of the trial your patients’ health state was:** | | | | |  |
| Improved | 48 | 35,8 | 29,6 | 20,7 | 31,2 |
| Unchanged | 103 | 64,2 | 70,5 | 69,0 | 66,9 |
| Deteriorated | 3 | 0,0 | 0,0 | 10,3 | 2,0 |
| **p-value : 0.0053** |  |  |  |  |  |
| **Viewpoints about cure trials** | | | | | |
| **In your opinion how important is it to be able to cure your patients of HIV?** | | | | | |
| Very important | 111 | 77,8 | 77,3 | 48,3 | 72,1 |
| Important | 38 | 22,2 | 22,7 | 34,5 | 24,7 |
| Not important | 5 | 0,0 | 0,0 | 17,2 | 3,3 |
| **p-value : <.0001** |  |  |  |  |  |
| **Do you think a cure treatment will become available during your career?** | | | | | |
| Yes | 54 | 32,1 | 54,6 | 13,8 | 35,1 |
| No | 66 | 42,0 | 27,3 | 69,0 | 42,9 |
| Do not know | 34 | 25,9 | 18,2 | 17,2 | 22,1 |
| **p-value : 0.0019** |  |  |  |  |  |
| **To participate in a cure trial there must be a lot of participating centers** | | | | | |
| Very important | 25 | 17,3 | 13,6 | 17,2 | 16,2 |
| Quite important | 70 | 51,9 | 38,6 | 37,9 | 45,5 |
| Not very important | 59 | 30,9 | 47,7 | 44,8 | 38,3 |
| **p-value : 0.3658** | |  |  |  |  |
| **To participate in a cure trial there must be a lot of participating patients** | | | | | |
| Very important | 40 | 30,9 | 20,5 | 20,7 | 26,0 |
| Quite important | 65 | 34,6 | 47,7 | 55,2 | 42,2 |
| Not very important | 49 | 34,6 | 31,8 | 24,1 | 31,8 |
| **p-value : 0.2981** | |  |  |  |  |
| **To participate in a cure trial there must be frequent examinations to verify patients’ health states** | | | | | |
| Very important | 87 | 46,9 | 79,6 | 48,3 | 56,5 |
| Quite important | 56 | 44,4 | 18,2 | 41,4 | 36,4 |
| Not very important | 11 | 8,6 | 2,3 | 10,3 | 7,1 |
| **p-value : 0.0087** |  |  |  |  |  |
| **To participate in a cure trial there must be a direct benefit for patients** | | | | | |
| Very important | 86 | 43,2 | 75,0 | 62,1 | 55,8 |
| Quite important | 52 | 43,2 | 25,0 | 20,7 | 33,8 |
| Not very important | 16 | 13,6 | 0,0 | 17,2 | 10,4 |
| **p-value : 0.0022** |  |  |  |  |  |
| **To participate in a cure trial molecules must already be tested for other diseases** | | | | | |
| Very important | 13 | 6,2 | 6,8 | 17,2 | 8,4 |
| Quite important | 41 | 22,2 | 40,9 | 17,2 | 26,6 |
| Not very important | 100 | 71,6 | 52,3 | 65,5 | 64,9 |
| **p-value : 0.0429** |  |  |  |  |  |
| **Agreeing with the “moderately motivated” viewpoint** | |  |  |  |  |
| <=25th | 49 | 32,1 | 25,0 | 41,4 | 31,8 |
| 25-75th | 49 | 34,6 | 31,8 | 24,1 | 31,8 |
| >=75th | 56 | 33,3 | 43,2 | 34,5 | 36,4 |
| p-value : 0.5573 | |  |  |  |  |
| **Agreeing with the “most motivated” viewpoint** | |  |  |  |  |
| <=25th | 55 | 27,2 | 40,9 | 51,7 | 35,7 |
| 25-75th | 30 | 13,6 | 34,1 | 13,8 | 19,5 |
| >=75th | 69 | 59,3 | 25,0 | 34,5 | 44,8 |
| p-value : 0.0007 |  |  |  |  |  |
| **Agreeing with the “benefit-centered” viewpoint** | |  |  |  |  |
| <=25th | 54 | 37,0 | 20,5 | 51,7 | 35,1 |
| 25-75th | 53 | 37,0 | 43,2 | 13,8 | 34,4 |
| >=75th | 47 | 25,9 | 36,4 | 34,5 | 30,5 |
| p-value : 0.0266 |  |  |  |  |  |
| **Agreeing with the “reticence and way of life” viewpoint** | |  |  |  |  |
| <=25th | 64 | 38,3 | 38,6 | 55,2 | 41,6 |
| 25-75th | 47 | 37,0 | 27,3 | 17,2 | 30,5 |
| >=75th | 43 | 24,7 | 34,1 | 27,6 | 27,9 |
| p-value : 0.2439 | |  |  |  |  |
| **Sources of information about HIV cure research** | | | | | |
| **International conferences** | | | | | |
| Yes | 103 | 74,1 | 50,0 | 72,4 | 66,9 |
| No | 51 | 25,9 | 50,0 | 27,6 | 33,1 |
| **p-value : 0.0187** |  |  |  |  |  |
| **National conferences** | | | | | |
| Yes | 133 | 88,9 | 81,8 | 86,2 | 86,4 |
| No | 21 | 11,1 | 18,2 | 13,8 | 13,6 |
| **p-value : 0.5458** | |  |  |  |  |
| **Meetings of learned societies** | | | | | |
| Yes | 104 | 71,6 | 61,4 | 65,5 | 67,5 |
| No | 50 | 28,4 | 38,6 | 34,5 | 32,5 |
| **p-value : 0.4892** | |  |  |  |  |
| **International scientific literature** | | | | | |
| Yes | 134 | 87,7 | 88,6 | 82,8 | 87,0 |
| No | 20 | 12,4 | 11,4 | 17,2 | 13,0 |
| **p-value : 0.7421** | |  |  |  |  |
| **Reviews from HIV associations** | | | | | |
| Yes | 68 | 55,6 | 22,7 | 44,8 | 44,2 |
| No | 86 | 44,4 | 77,3 | 55,2 | 55,8 |
| **p-value : 0.0020** |  |  |  |  |  |
| **websites** | | | | | |
| Yes | 91 | 53,1 | 59,1 | 75,9 | 59,1 |
| No | 63 | 46,9 | 40,9 | 24,1 | 40,9 |
| **p-value : 0.1011** | |  |  |  |  |
| **In your opinion, how concerned are your patients about the following negative aspects of HIV** | | | | | |
| **Shorter expected life** | | | | | |
| Very concerned | 28 | 19,8 | 15,9 | 17,2 | 18,2 |
| A little concerned | 84 | 66,7 | 45,5 | 34,5 | 54,6 |
| Not at all concerned | 42 | 13,6 | 38,6 | 48,3 | 27,3 |
| **p-value : 0.0015** |  |  |  |  |  |
| **Severe fatigue** | | | | | |
| Very concerned | 21 | 19,8 | 6,8 | 6,9 | 13,6 |
| A little concerned | 93 | 59,3 | 65,9 | 55,2 | 60,4 |
| Not at all concerned | 40 | 21,0 | 27,3 | 37,9 | 26,0 |
| **p-value : 0.1136** | |  |  |  |  |
| **A negative impact on health** | | | | | |
| Very concerned | 60 | 46,9 | 34,1 | 24,1 | 39,0 |
| A little concerned | 76 | 45,7 | 59,1 | 44,8 | 49,4 |
| Not at all concerned | 18 | 7,4 | 6,8 | 31,0 | 11,7 |
| **p-value : 0.0032** |  |  |  |  |  |
| **An uncertain future** | | | | | |
| Very concerned | 62 | 46,9 | 40,9 | 20,7 | 40,3 |
| A little concerned | 70 | 44,4 | 45,5 | 48,3 | 45,5 |
| Not at all concerned | 22 | 8,6 | 13,6 | 31,0 | 14,3 |
| **p-value : 0.0240** |  |  |  |  |  |
| **The risk of transmitting the disease** | | | | | |
| Very concerned | 85 | 60,5 | 52,3 | 44,8 | 55,2 |
| A little concerned | 55 | 35,8 | 45,5 | 20,7 | 35,7 |
| Not at all concerned | 14 | 3,7 | 2,3 | 34,5 | 9,1 |
| **p-value : <.0001** |  |  |  |  |  |
| **Discrimination** | | |  |  |  |
| Very concerned | 131 | 93,8 | 77,3 | 72,4 | 85,1 |
| A little or not concerned | 23 | 6,2 | 22,7 | 27,6 | 14,9 |
| **p-value : 0.0049** |  |  |  |  |  |
| **Difficulties constructing a stable couple relationship** | | | |  |  |
| Very concerned | 129 | 88,9 | 84,1 | 69,0 | 83,8 |
| A little or not concerned | 25 | 11,1 | 15,9 | 31,0 | 16,2 |
| **p-value : 0.0442** |  |  |  |  |  |
| **Having to live with a secret** | | |  |  |  |
| Very concerned | 133 | 92,6 | 93,2 | 58,6 | 86,4 |
| A little or not concerned | 21 | 7,4 | 6,8 | 41,4 | 13,6 |
| **p-value : <.0001** |  |  |  |  |  |
| **Feeling unable to lead a normal life** | | | | | |
| Very concerned | 67 | 55,6 | 40,9 | 13,8 | 43,5 |
| A little concerned | 75 | 38,3 | 56,8 | 65,5 | 48,7 |
| Not at all concerned | 12 | 6,2 | 2,3 | 20,7 | 7,8 |
| **p-value : 0.0003** |  |  |  |  |  |
| **A negative impact on sexuality** | | |  |  |  |
| Very concerned | 125 | 86,4 | 86,4 | 58,6 | 81,2 |
| A little or not concerned | 29 | 13,6 | 13,6 | 41,4 | 18,8 |
| **p-value : 0.0026** |  |  |  |  |  |
| **Having to use condoms** | | | | | |
| Very concerned | 76 | 54,3 | 43,2 | 44,8 | 49,4 |
| A little concerned | 68 | 43,2 | 52,3 | 34,5 | 44,2 |
| Not at all concerned | 10 | 2,5 | 4,6 | 20,7 | 6,5 |
| **p-value : 0.0094** |  |  |  |  |  |
| **A negative impact on professional lives** | | | | | |
| Very concerned | 28 | 21,0 | 20,5 | 6,9 | 18,2 |
| A little concerned | 101 | 70,4 | 61,4 | 58,6 | 65,6 |
| Not at all concerned | 25 | 8,6 | 18,2 | 34,5 | 16,2 |
| **p-value : 0.0170** |  |  |  |  |  |
| **The cost for society** | | | | | |
| Very concerned | 40 | 22,2 | 29,6 | 31,0 | 26,0 |
| A little concerned | 72 | 55,6 | 38,6 | 34,5 | 46,8 |
| Not at all concerned | 42 | 22,2 | 31,8 | 34,5 | 27,3 |
| **p-value : 0.2414** | |  |  |  |  |
| **Out-of-pocket expenses** | | | | | |
| Very concerned | 22 | 13,6 | 25,0 | 0,0 | 14,3 |
| A little concerned | 80 | 54,3 | 52,3 | 44,8 | 52,0 |
| Not at all concerned | 52 | 32,1 | 22,7 | 55,2 | 33,8 |
| **p-value : 0.0084** |  |  |  |  |  |
| **Perception of life with ART for your patients** | | | | | |
| **Overall, how would you say your patients feel about having to take ARV treatment?** | | | | | |
| Very comfortable | 13 | 6,2 | 9,1 | 13,8 | 8,4 |
| Comfortable | 135 | 93,8 | 79,6 | 82,8 | 87,7 |
| Uncomfortable | 6 | 0,0 | 11,4 | 3,5 | 3,9 |
| **p-value : 0.0195** |  |  |  |  |  |
| **Do you think it is easy for your patients to take ART every day?** | | | | | |
| **Yes** | 75 | 37,0 | 56,8 | 69,0 | 48,7 |
| **No** | 79 | 63,0 | 43,2 | 31,0 | 51,3 |
| **p-value : 0.0057** |  |  |  |  |  |
| **How do you think your patients are feeling regarding side effects related to ART?** | | | | | |
| Very concerned | 74 | 59,3 | 43,2 | 24,1 | 48,1 |
| A little or not concerned | 80 | 40,7 | 56,8 | 75,9 | 52,0 |
| **p-value : 0.0038** |  |  |  |  |  |
| **In your opinion, how uncomfortable are ART-related side effects for your patients?** | | | | | |
| Very uncomfortable | 22 | 17,3 | 9,1 | 13,8 | 14,3 |
| A little uncomfortable | 120 | 80,3 | 84,1 | 62,1 | 77,9 |
| Not at all uncomfortable | 12 | 2,5 | 6,8 | 24,1 | 7,8 |
| **p-value : 0.0038** |  |  |  |  |  |
| **Confidence in current ART** | | | | |  |
| **Are you very confident with the current ART?** | | | | |  |
| No | 70 | 44,4 | 54,6 | 34,5 | 45,5 |
| Yes | 84 | 55,6 | 45,5 | 65,5 | 54,6 |
| **p-value : 0.2336** | |  |  |  |  |
| **Do you think the current ART will continue to be effective in the long term?** | | | | |  |
| Yes | 137 | 88,9 | 93,2 | 82,8 | 89,0 |
| No | 17 | 11,1 | 6,8 | 17,2 | 11,0 |
| **p-value : 0.3801** | |  |  |  |  |
