# Supplementary material for: Differences in HIV cure clinical trial preferences of French people living with HIV and physicians in the ANRS‐APSEC study: a discrete choice experiment
Source: J Int AIDS Soc. 2020 Feb 20;23(2):e25443. doi: 10.1002/jia2.25443 (PMC7048214; doi:10.1002/jia2.25443)
Supplement: Supplementary file 3 — Table S3. Description of four HCRCT candidates according to attribute levels [file JIA2-23-e25443-s003.docx]

# Table S3. Description of four HCRCT candidates according to attribute levels

|  | Attribute levels | | | |
| --- | --- | --- | --- | --- |
| Attributes | **Latency reactivation** | **Immunotherapy** | **Gene therapy** | **Combined therapy** (LR + Immuno) |
| **Trial duration** | 6-9 months | 15-18 months | 15-18 months | 15-18 months |
| **Consultation frequency** | Weekly | Monthly | Weekly | Weekly |
| **Moderate SE**  (1-10%, few days) | Digestive disorders | Flu-type syndrome | Digestive disorders | Digestive disorders, Flu-type syndrome, fatigue |
| **Severe SE**  (<1/10000) | Allergy, infections | Allergy | Allergy, infections, risk of cancer a few years later^$^ | Allergy, infections |
| **Outcomes**  (ATI duration, % of success) | 3-6 months, 5% | 3-6 months, 5% | 6-12 months, 10% | 6-12 months, 10% |

$: missing data for estimating risk frequency.
