# Supplementary material for: Differences in HIV cure clinical trial preferences of French people living with HIV and physicians in the ANRS‐APSEC study: a discrete choice experiment
Source: J Int AIDS Soc. 2020 Feb 20;23(2):e25443. doi: 10.1002/jia2.25443 (PMC7048214; doi:10.1002/jia2.25443)
Supplement: Supplementary file 4 — Table S4. Detailed results of the discrete choice experiment [file JIA2-23-e25443-s004.docx]

# Table S4. Detailed results of the mixed (random effects) logit models with correlated coefficients.

## DCE results for the PLWH (n=195)

| Attribute/Level | Mean (SE) | *p* | SD^[[1]](#footnote-1)^ (SE) | *p* |  |
| --- | --- | --- | --- | --- | --- |
| Intercept | 0,16 (0.10) | 0.107 | 0.08 (0.13) | 0.58 |  |
| **Trial Duration** (ref. 15-18 months) | | | | |  |
| 6-9 months | 0.79 (0.16) | *** | 0.96 (0.18) | *** |  |
| **Consultation frequency** (ref. weekly) | | | | |  |
| Monthly | 2.50 (0.29) | *** | 2.79 (0.30) | *** |  |
| **Moderate side effects** (ref. Flu-type syndrome, digestive disorders, fatigue) | | | | |  |
| Digestive disorders | 1.90 (0.30) | *** | 1.49 (0.26) | *** |  |
| Flu-type syndrome | 1.34 (0.29) | *** | 1.52 (0.32) | *** |  |
| **Severe side effects** (ref. Allergy, infections, risk of cancer ) | | | | |  |
| Allergy | 4.52 (0.50) | *** | 3.84 (0.42) | *** |  |
| Allergy, infections | 3.87 (0.44) | *** | 2.72 (0.36) | *** |  |
| **Outcomes** (ref. 3-6 months, 5%) | | | | |  |
| 6-12 months, 10% | 1.14 (0.22) | *** | 1.59 (0.24) | *** |  |
| Log-likelihood = -1084.5; AIC = 2257.0 | | | | |  |
| Abbreviations: SD=standard deviation; SE= standard error. * *p*≤0.05; ** *p*≤0.01; *** *p*≤0.001 | | | | | |

## DCE results for the physicians (n=160)

| Attribute/Level | Mean (SE) | p | SD (SE) | p |
| --- | --- | --- | --- | --- |
| Intercept | 0.04 (0.22) | 0.84 | 1.44 (0.38) | *** |
| **Trial Duration** (ref. 15-18 months) | | | | |
| 6-9 months | 0.16 (0.26) | 0.56 | 1.70 (0.39) | *** |
| **Consultation frequency** (ref. weekly) | | | | |
| Monthly | 4.95 (0.80) | *** | 4.22 (0.74) | *** |
| **Moderate side effects** (ref. Flu-type syndrome, digestive disorders, fatigue) | | | | |
| Digestive disorders | 3.12 (0.67) | *** | 2.33 (0.67) | *** |
| Flu-type syndrome | 0.94 (0.53) | 0.07 | 3.69 (0.75) | *** |
| **Severe side effects** (ref. Allergy, infections, risk of cancer ) | | | | |
| Allergy | 10.54 (1.71) | *** | 6.95 (1.25) | *** |
| Allergy, infections | 10.12 (1.67) | *** | 5.68 (1.02) | *** |
| **Outcomes** (ref. 3-6 months, 5%) | | | | |
| 6-12 months, 10% | 6.77 (1.18) | *** | 4.75 (1.00) | *** |
| Log-likelihood = -731.9; AIC = 1551.8 |  |  |  |  |
| Abbreviations: SD=standard deviation; SE= standard error. * p≤0.05; ** p≤0.01; *** p≤0.001 | | | | |

## DCE results for the three PLWH profiles (MXL with interactions)

### Comfortable & confident PLWH profile (profile 1 vs. profiles 2&3) (n=195)

| Attribute/Level | Mean (SE) | *p* | SD (SE) | *p* |  |
| --- | --- | --- | --- | --- | --- |
| Intercept | 0.26 (0.13) | * | 0.11 (0.17) | 0.50 | |
| Intercept * Profile 1 (ref. Profiles 2&3) | 0.00 (0.18) | 0.99 |  |  | |
| **Trial Duration** (ref. 15-18 months) | | | | |  |
| 6-9 months | 0.75 (0.23) | *** | 1.22 (0.27) | *** |  |
| 6-9 months * Profile 1 (ref. 15-18 months, Profiles 2&3) | 0.19 (0.32) | 0.55 |  |  |  |
| **Consultations frequency** (ref. weekly) | | | | |  |
| Monthly | 2.41 (0.41) | *** | 2.86 (0.35) | *** |  |
| Monthly * Profile 1 (ref. Weekly, Profiles 2&3) | 0.88 (0.59) | 0.14 |  |  |  |
| **Moderate side effects** (ref. Flu-type syndrome, digestive disorders, fatigue) | | | | |  |
| Digestive disorders | 1.91 (0.36) | *** | 1.88 (0.32) | *** |  |
| Digestive disorders * Profile 1 (ref. Flu-type syndrome, digestive disorders, fatigue, Profiles 2&3) | 0.25 (0.43) | 0.56 |  |  |  |
| Flu-type syndrome | 1.23 (0.40) | ** | 2.26 (0.42) | *** |  |
| Flu-type syndrome * Profile 1 (ref. Flu-type syndrome, digestive disorders, fatigue, Profiles 2&3) | 0.05 (0.48) | 0.92 |  |  |  |
| **Severe side effects** (ref. Allergy, infections, risk of cancer) | | | | |  |
| Allergy | 4.57 (0.67) | *** | 4.68 (0.62) | *** |  |
| Allergy * Profile 1 (ref. Allergy, infections, risk of cancer, Profiles 2&3) | 0.15 (0.74) | 0.84 |  |  |  |
| Allergy, infections | 4.08 (0.54) | *** | 3.02 (0.45) | *** |  |
| Allergy, infections * Profile 1 (ref. Allergy, infections, risk of cancer, Profiles 2&3) | -0.02 (0.60) | 0.97 |  |  |  |
| **Outcomes** (ref. 3-6 months, 5%) | | | | |  |
| 6-12 months, 10% | 1.00 (0.28) | *** | 1.75 (0.29) | *** |  |
| 6-12 months, 10%* Profile 1 (ref. 3-6 months, 5%, Profiles 2&3) | 0.57 (0.37) | 0.12 |  |  |  |
| Log-likelihood =-1079.3 ; AIC = 2262.5 |  |  |  |  |  |
| Abbreviations: SD=standard deviation; SE= standard error. * *p*≤0.05; ** *p*≤0.01; *** *p*≤0.001 | | | | |  |

### Moderate PLWH profile (profile 2 vs. profiles1&3) (n=195)

| Attribute/Level | Mean (SE) | *p* | SD (SE) | *p* |
| --- | --- | --- | --- | --- |
| Intercept | 0.07 (0.11) | 0.49 | 0.07 (0.13) | 0.59 |
| Intercept * Profile 2 (ref. Profiles 1&3) | 0.40 (0.21) | 0.06 |  |  |
| **Trial Duration** (ref. 15-18 months) | | | | |
| 6-9 months | 0.71 (0.17) | *** | 0.97 (0.18) | *** |
| 6-9 months * Profile 2 (ref. 15-18 months, Profiles 1&3) | 0.47 (0.32) | 0.14 |  |  |
| **Consultation frequency** (ref. weekly) | | | | |
| Monthly | 2.57 (0.32) | *** | 2.84 (0.31) | *** |
| Monthly * Profile 2 (ref. weekly, Profiles 1&3) | -0.11 (0.49) | 0.83 |  |  |
| **Moderate side effects** (ref. Flu-type syndrome, digestive disorders, fatigue) | | | | |
| Digestive disorders | 1.87 (0.32) | *** | 1.52 (0.26) | *** |
| Digestive disorders * Profile 2 (ref. Flu-type syndrome, digestive disorders, fatigue ,Profiles 1&3) | 0.25 (0.47) | 0.59 |  |  |
| Flu-type syndrome | 1.37 (0.31) | *** | 1.53 (0.32) | *** |
| Flu-type syndrome * Profile 2 (ref. Flu-type syndrome, digestive disorders, fatigue, Profiles 1&3) | -0.13 (0.48) | 0.78 |  |  |
| **Severe side effects** (ref. Allergy, infections, risk of cancer) | | | | |
| Allergy | 4.58 (0.52) | *** | 3.89 (0.43) | *** |
| Allergy * Profile 2 (ref. Allergy, infections, risk of cancer, Profiles 1&3) | -0.04 (0.70) | 0.96 |  |  |
| Allergy, infections | 3.94 (0.46) | *** | 2.75 (0.36) | *** |
| Allergy, infections * Profile 2 (ref. Allergy, infections, risk of cancer, Profiles 1&3) | -0.03 (0.62) | 0.96 |  |  |
| **Outcomes** (ref. 3-6 months, 5%) | | | | |
| 6-12 months, 10% | 1.21 (0.25) | *** | 1.63 (0.24) | *** |
| 6-12 months, 10%* Profile 2 (ref. 3-6 months, 5%, Profiles 1&3) | -0.22 (0.43) | 0.62 |  |  |
| Log-likelihood = -1079.9; AIC = 2263.7 |  |  |  |  |
| Abbreviations: SD=standard deviation; SE= standard error. * *p*≤0.05; ** *p*≤0.01; *** *p*≤0.001 | | | | |

### Vulnerable & unconfident PLWH profile (profile 3 vs. profiles 1&2) (n=195)

| Attribute/Level | Mean (SE) | *p* | SD (SE) | *p* |
| --- | --- | --- | --- | --- |
| Intercept | 0.36 (0.12) | ** | 0.08 (0.18) | 0.65 |
| Intercept * Profile 3 (ref. Profiles 1&2) | -0.30 (0.19) | 0.11 |  |  |
| **Trial Duration** (ref. 15-18 months) | | | | |
| 6-9 months | 0.98 (0.21) | *** | 1.17 (0.23) | *** |
| 6-9 months * Profile 3 (ref. 15-18 months, Profiles 1&2) | -0.35 (0.32) | 0.27 |  |  |
| **Consultation frequency** (ref. weekly) | | | | |
| Monthly | 3.06 (0.42) | *** | 2.80 (0.34) | *** |
| Monthly * Profile 3 (ref. Weekly, Profiles 1&2) | -0.68 (0.50) | 0.18 |  |  |
| **Moderate side effects** (ref. Flu-type syndrome, digestive disorders, fatigue) | | | | |
| Digestive disorders | 2.00 (0.39) | *** | 1.88 (0.35) | *** |
| Digestive disorders * Profile 3 (ref. Flu-type syndrome, digestive disorders, fatigue, Profiles 1&2) | -0.19 (0.44) | 0.66 |  |  |
| Flu-type syndrome | 1.04 (0.39) | ** | 2.27 (0.46) | *** |
| Flu-type syndrome * Profile 3 (ref. Flu-type syndrome, digestive disorders, fatigue, Profiles 1&2) | 0.26 (0.48) | 0.59 |  |  |
| **Severe side effects** (ref. Allergy, infections, risk of cancer) | | | | |
| Allergy | 4.29 (0.62) | *** | 4.62 (0.59) | *** |
| Allergy * Profile 3 (ref. Allergy, infections, risk of cancer, Profiles 1&2) | 0.48 (0.70) | 0.49 |  |  |
| Allergy, infections | 3.85 (0.57) | *** | 2.99 (0.44) | *** |
| Allergy, infections * Profile 3 (ref. Allergy, infections, risk of cancer, Profiles 1&2) | 0.45 (0.60) | 0.46 |  |  |
| **Outcomes** (ref. 3-6 months, 5%) | | | | |
| 6-12 months, 10% | 1.40 (0.28) | *** | 1.74 (0.29) | *** |
| 6-12 months, 10%* Profile 3 (ref. 3-6 months, 5%, Profiles 1&2) | -0.37 (0.40) | 0.35 |  |  |
| Log-likelihood = -1079.1; AIC = 2262.2 |  |  |  |  |
| Abbreviations: SD=standard deviation; SE= standard error. * *p*≤0.05; ** *p*≤0.01; *** *p*≤0.001 | | | | |

For each of the three PLWH intra-group profiles, estimates of means were summed across preference weights (global and interaction estimates for each level of each attribute) in order to obtain an estimation of the mean preference weight specific to each PLWH profile. Standard errors and p-values of these new parameters were computed using the delta method. Summed parameters were used for the calculation of the utilities associated with the four specific HCRCT.

### Summarized results for the DCE for the three PLWH profiles (sum of global and interaction estimates)

|  | ***Comfortable & confident*** | | | ***Moderate*** | | | | ***Vulnerable & unconfident*** | | |
| --- | --- | --- | --- | --- | --- | --- | --- | --- | --- | --- |
| Attribute/Level | β | SE | p | β | SE | p | β | | SE | p |
| Intercept | 0.26 | 0.14 | 0.071 | 0.48 | 0.20 | 0.015 | 0.05 | | 0.16 | 0.738 |
| **Trial Duration** (ref. 15-18 months) | | | | | | | | | | |
| 6-9 months | 0.95 | 0.24 | 0.000 | 1.18 | 0.31 | 0.000 | 0.63 | | 0.27 | 0.018 |
| **Consultation frequency** (ref. weekly) | | | | | | | | | | |
| Monthly | 3.29 | 0.47 | 0.000 | 2.47 | 0.49 | 0.000 | 2.38 | | 0.45 | 0.000 |
| **Moderate side effects** (ref. Flu-type syndrome, digestive disorders, fatigue) | | | | | | | | | | |
| Digestive disorders | 2.16 | 0.45 | 0.000 | 2.12 | 0.49 | 0.000 | 1.81 | | 0.41 | 0.000 |
| Flu-type syndrome | 1.29 | 0.44 | 0.004 | 1.24 | 0.48 | 0.010 | 1.30 | | 0.43 | 0.003 |
| **Severe side effects** (ref. Allergy, infections, risk of cancer) | | | | | | | | | | |
| Allergy | 4.72 | 0.73 | 0.000 | 4.54 | 0.75 | 0.000 | 4.77 | | 0.71 | 0.000 |
| Allergy, infections | 4.06 | 0.61 | 0.000 | 3.90 | 0.67 | 0.000 | 4.29 | | 0.61 | 0.000 |
| **Outcomes** (ref. 3-6 months, 5%) | | | | | | | | | | |
| 6-12 months, 10% | 1.57 | 0.32 | 0.000 | 0.99 | 0.40 | 0.013 | 1.02 | | 0.38 | 0.007 |

|

## DCE results for the three physician profiles (MXL with interactions)

### Engaged & patient-centered physician profile (profile 1 vs. profiles 2&3) (n=160)

| Attribute/Level | Mean (SE) | *p* | SD (SE) | *p* |
| --- | --- | --- | --- | --- |
| Intercept | -0.15 (0.31) | 0.63 | 1.57 (0.38) | *** |
| Intercept * Profile 1 (ref. Profiles 2&3) | 0.66 (0.44) | 0.13 |  |  |
| **Trial Duration** (ref. 15-18 months) |  |  |  |  |
| 6-9 months | 0.27 (0.40) | 0.50 | 1.96 (0.49) | *** |
| 6-9 months * Profile 1 (ref. 15-18 months, Profiles 2&3) | 0.24 (0.59) | 0.68 |  |  |
| **Consultation frequency** (ref. weekly) |  |  |  |  |
| Monthly | 5.05 (0.95) | *** | 5.02 (1.02) | *** |
| Monthly * Profile 1 (ref. Weekly, Profiles 2&3) | 1.85 (0.69) | ** |  |  |
| **Moderate side effects** (ref. Flu-type syndrome, digestive disorders, fatigue) |  |  |  |  |
| Digestive disorders | 2.78 (0.81) | *** | 2.66 (0.84) | ** |
| Digestive disorders * Profile 1 (ref. Flu-type syndrome, digestive disorders, fatigue, Profiles 2&3) | 2.26 (1.01) | * |  |  |
| Flu-type syndrome | 0.61 (0.63) | 0.34 | 4.29 (1.11) | *** |
| Flu-type syndrome * Profile 1 (ref. Flu-type syndrome, digestive disorders, fatigue, Profiles 2&3) | 1.87 (1.00) | 0.06 |  |  |
| **Severe side effects** (ref. Allergy, infections, risk of cancer) |  |  |  |  |
| Allergy | 8.94 (1.76) | *** | 7.68 (1.64) | *** |
| Allergy * Profile 1 (réf. Allergy, infections, risk of cancer, Profiles 2&3) | 6.50 (1.77) | *** |  |  |
| Allergy. infections | 8.43 (1.79) | *** | 6.35 (1.47) | *** |
| Allergy. infections * Profile 1 (ref. Allergy, infections, risk of cancer, Profiles 2&3) | 6.09 (1.62) | *** |  |  |
| **Outcomes** (ref. 3-6 months, 5%) |  |  |  |  |
| 6-12 months. 10% | 5.28 (1.15) | *** | 5.10 (1.10) | *** |
| 6-12 months. 10%* Profile 1 (ref. 3-6 months, 5%, Profiles 2&3) | 4.17 (0.97) | *** |  |  |
| Log-likelihood = -715.31 ; AIC = 1534.6 |  |  |  |  |
| Abbreviations: SD=standard deviation; SE= standard error. * *p*≤0.05; ** *p*≤0.01; *** *p*≤0.001 |  |  |  |  |

### Least experienced & moderate physician profile (profile 2 vs. profiles 1&3) (n=160)

| Attribute/Level | Mean (SE) | *p* | SD (SE) | *p* |
| --- | --- | --- | --- | --- |
| Intercept | 0.58 (0.29) | * | 1.69 (0.35) | *** |
| Intercept * Profile 2 (ref. Profiles 1&3) | -0.97 (0.43) | * |  |  |
| **Trial Duration** (ref. 15-18 months) |  |  |  |  |
| 6-9 months | 1.23 (0.44) | ** | 2.06 (0.44) | *** |
| 6-9 months * Profile 2 (ref. 15-18 months. Profiles 1&3) | -0.78 (0.53) | 0.14 |  |  |
| **Consultation frequency** (ref. weekly) |  |  |  |  |
| Monthly | 8.59 (1.34) | *** | 5.50 (0.86) | *** |
| Monthly * Profile 2 (ref. Weekly. Profiles 1&3) | -3.49 (0.76) | *** |  |  |
| **Moderate side effects** (ref. Flu-type syndrome, digestive disorders, fatigue) |  |  |  |  |
| Digestive disorders | 5.38 (1.15) | *** | 2.97 (0.77) | *** |
| Digestive disorders * Profile 2 (ref. Flu-type syndrome, digestive disorders, fatigue. Profiles 1&3) | -1.60 (0.83) | 0.06 |  |  |
| Flu-type syndrome | 2.22 (0.89) | * | 4.97 (0.96) | *** |
| Flu-type syndrome * Profile 2 (ref. Flu-type syndrome, digestive disorders, fatigue. Profiles 1&3) | -0.69 (1.01) | 0.50 |  |  |
| **Severe side effects** (ref. Allergy, infections, risk of cancer) |  |  |  |  |
| Allergy | 15.05 (2.36) | *** | 8.44 (1.40) | *** |
| Allergy * Profile 2 (réf. Allergy, infections, risk of cancer. Profiles 1&3) | -3.40 (1.13) | ** |  |  |
| Allergy. infections | 15.46 (2.55) | *** | 7.46 (1.42) | *** |
| Allergy. infections * Profile 2 (ref. Allergy, infections, risk of cancer. Profiles 1&3) | -5.95 (1.41) | *** |  |  |
| **Outcomes** (ref. 3-6 months. 5%) |  |  |  |  |
| 6-12 months. 10% | 9.55 (1.50) | *** | 5.18 (0.84) | *** |
| 6-12 months. 10%* Profile 2 (ref. 3-6 months. 5%. Profiles 1&3) | -3.69 (0.97) | *** |  |  |
| Log-likelihood = -714.7; AIC = 1533.3 |  |  |  |  |
| Abbreviations: SD=standard deviation; SE= standard error. * *p*≤0.05; ** *p*≤0.01; *** *p*≤0.001 |  |  |  |  |

### Most experienced & reticent physician profile (profile 3 vs. profiles 1&2) (n=160)

| Attribute/Level | Mean (SE) | *p* | SD (SE) | *p* |
| --- | --- | --- | --- | --- |
| Intercept | 0.38 (0.24) | 0.11 | 1.28 (0.26) | *** |
| Intercept * Profile 3 (ref. Profiles 1&2) | -0.30 (0.39) | 0.45 |  |  |
| **Trial Duration** (ref. 15-18 months) |  |  |  |  |
| 6-9 months | 0.34 (0.29) | 0.24 | 1.71 (0.35) | *** |
| 6-9 months * Profile 3 (ref. 15-18 months, Profiles 1&2) | 0.07 (0.53) | 0.90 |  |  |
| **Consultation frequency** (ref. weekly) |  |  |  |  |
| Monthly | 4.84 (0.71) | *** | 3.75 (0.54) | *** |
| Monthly * Profile 3 (ref. Weekly, Profiles 1&2) | -0.10 (0.68) | 0.89 |  |  |
| **Moderate side effects** (ref. Flu-type syndrome, digestive disorders, fatigue) |  |  |  |  |
| Digestive disorders | 3.27 (0.68) | *** | 1.89 (0.52) | *** |
| Digestive disorders * Profile 3 (ref. Flu-type syndrome, digestive disorders, fatigue. Profiles 1&2) | -0.62 (0.82) | 0.45 |  |  |
| Flu-type syndrome | 1.09 (0.53) | * | 3.35 (0.64) | *** |
| Flu-type syndrome * Profile 3 (ref. Flu-type syndrome, digestive disorders, fatigue, Profiles 1&2) | 0.16 (0.85) | 0.85 |  |  |
| **Severe side effects** (ref. Allergy, infections. risk of cancer) |  |  |  |  |
| Allergy | 10.82 (1.49) | *** | 6.17 (0.95) | *** |
| Allergy * Profile 3 (ref. Allergy, infections. risk of cancer, Profiles 1&2) | -3.71 (1.47) | * |  |  |
| Allergy. infections | 10.14 (1.56) | *** | 5.00 (0.82) | *** |
| Allergy. infections * Profile 3 (ref. Allergy, infections. risk of cancer, Profiles 1&2) | -2.27 (1.35) | 0.09 |  |  |
| **Outcomes** (ref. 3-6 months, 5%) |  |  |  |  |
| 6-12 months, 10% | 6.71 (0.95) | *** | 3.97 (0.64) | *** |
| 6-12 months, 10%* Profile 3 (ref. 3-6 months, 5%, Profiles 1&2) | -2.68 (0.85) | ** |  |  |
| Log-likelihood = -721.0; AIC = 1546.0 |  |  |  |  |
| Abbreviations: SD=standard deviation; SE= standard error. * *p*≤0.05; ** *p*≤0.01; *** *p*≤0.001 |  |  |  |  |

### Summarized results for the DCE for the three physician profiles (sum of the global and interaction estimates)

|  | ***Engaged & patient-centered*** | | | | ***Least experienced & moderate*** | | | | ***Most experienced & reticent*** | | | |
| --- | --- | --- | --- | --- | --- | --- | --- | --- | --- | --- | --- | --- |
| Attribute/Level | β | SE | p | β | | SE | p | β | | SE | p |  |
| Intercept | 0.51 | 0.33 | 0.120 | -0.39 | | 0.34 | 0.246 | 0.08 | | 0.34 | 0.813 |  |
| **Trial Duration** (ref. 15-18 months) | | | | | | | | | | | | |
| 6-9 months | 0.51 | 0.48 | 0.284 | 0.45 | | 0.43 | 0.289 | 0.41 | | 0.48 | 0.395 |  |
| **Consultation frequency** (ref. weekly) | | | | | | | | | | | |  |
| Monthly | 6.91 | 1.36 | 0.000 | 5.10 | | 1.02 | 0.000 | 4.74 | | 0.85 | 0.000 |  |
| **Moderate side effects** (ref. Flu-type syndrome, digestive disorders, fatigue) | | | | | | | | | | | | |
| Digestive disorders | 5.03 | 1.37 | 0.000 | 3.78 | | 1.07 | 0.000 | 2.65 | | 0.97 | 0.007 |  |
| Flu-type syndrome | 2.48 | 1.14 | 0.030 | 1.53 | | 0.93 | 0.099 | 1.26 | | 0.90 | 0.162 |  |
| **Severe side effects** (ref. Allergy, infections, risk of cancer) | | | | | | | | | | | | |
| Allergy | 15.45 | 3.21 | 0.000 | 11.64 | | 2.05 | 0.000 | 7.10 | | 1.69 | 0.000 |  |
| Allergy, infections | 14.52 | 3.09 | 0.000 | 9.52 | | 1.82 | 0.000 | 7.87 | | 1.62 | 0.000 |  |
| **Outcomes** (ref. 3-6 months, 5%) | | | | | | | | | | | | |
| 6-12 months, 10% | 9.45 | 1.70 | 0.000 | 5.86 | | 1.09 | 0.000 | 4.03 | | 0.85 | 0.000 |  |

1. : The standard deviation (SD) for each level of a given attribute represents how different the weight associated with that level is across respondents. Larger (smaller) standard deviations indicate greater (lesser) variability in the weight associated with that attribute level. [↑](#footnote-ref-1)
